# High Electron Charge Carrier Mobility in the Nematic Phase of a Roof‐Shaped Nematogen with Optimum Molecular Biaxiality

**DOI:** 10.1002/advs.202510009

**Published:** 2025-07-25

**Authors:** Matthias Lehmann, Nikolai Scheuring, Loïc Mager, Dharmendra Pratap Singh, Richard Mandle, Alexey Eremin

**Affiliations:** ^1^ Institute of Organic Chemistry University of Würzburg Am Hubland 97074 Würzburg Germany; ^2^ Center of Nanosystems Chemistry and Bavarian Polymer Institute Theodor‐Bovori‐Weg 4 97074 Würzburg Germany; ^3^ Institut de Physique et Chimie des Matériaux de Strasbourg BP 43, 67034, Cedex 2 Strasbourg France; ^4^ Unité de Dynamique et Structure des Matériaux Moléculaires (UDSMM) Université du Littoral Côte d'Opale (ULCO) 50 Rue Ferdinand Buisson Calais 62100 France; ^5^ School of Physics and Astronomy School of Chemistry University of Leeds Leeds LS2 9JT England; ^6^ Institute of Physics Otto von Guericke University 39106 Magdeburg Germany

**Keywords:** liquid crystal, molecular biaxiality, nematic, n‐type material, TOF mobility

## Abstract

A roof‐shaped molecule forming exclusively a nematic liquid crystal phase is prepared based on a lead structure. The aspect ratio is designed to be almost optimum with respect to the molecular biaxiality. A broad nematic phase over more than 100 K is observed in which a weak, transient biaxial alignment can be induced under specific thermal and mechanical conditions. In sandwich cells, TOF electron mobilities of up to 2.2 × 10^−2^ cm^2 ^V^−1 ^s^−1^ are obtained, which are the highest found to date for nematic materials. The latter is attributed to the special self‐assembly of the biaxial, roof‐shaped mesogens, which favors the contacts between the aromatic units, which is confirmed by X‐ray scattering, modeling, and X‐ray scattering simulation. The material is responsive to the applied electric field, which results in the anomalous negative field dependence of the charge carrier mobilities.

## Graphical abstract

Liquid Roofs. Biaxial roof‐shaped mesogens self‐assemble in a nematic phase of a broad temperature range exhibiting high TOF electron mobilities. These mesogens possess the almost optimum biaxial aspect ratio. The results from X‐ray, polarized optical microscopy, modeling, and TOF data agree with biaxiality of small domains and transient nematic phase biaxiality.

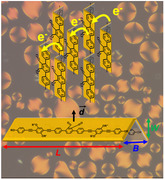

## 1 Introduction

Soft functional materials are the focus of research owing to the possibility to tailor their properties by synthetic procedures, their flexibility, and their stimuli responsiveness.^[^ ^1^, ^2^ ^]^ One of the low molar mass materials is liquid crystals (LCs), which have been applied in information technology as switches and in the display industry as light valves.^[^ ^3^ ^]^ New applications are emerging in which LCs are used as charge transport materials in devices such as photovoltaic cells, field‐effect transistors, or light‐emitting diodes.^[^ ^4^, ^5^, ^6^, ^7^, ^8^ ^]^ Record charge carrier mobilities are reported for highly ordered soft crystalline structures obtained from 1D columnar or 2D lamellar self‐assemblies.^[^ ^8^, ^9^, ^10^ ^]^ However, these systems require a precise alignment, which is not always easy to obtain. Therefore, semiconductors with nematic LC phases were also investigated, since for such phases, alignment layers are industrially optimised for planar or homeotropic alignment, and the comparable low viscosity of the LC phase allows an easy orientation in magnetic and electric fields. Thus, molecular engineering for low clearing temperatures can be avoided, which is necessary to align higher ordered phases by cooling from the isotropic liquid. However, low molar mass and low viscosity nematogens lack high charge carrier mobility, and seldom charge carrier mobilities above 10^−3^ cm^2^ V^−1^s^−1^ are achieved.^[^ ^11^, ^12^, ^13^, ^14^, ^15^, ^16^, ^17^, ^18^, ^19^ ^]^ Nevertheless, for such materials, the facile construction of working printable electronic devices has been demonstrated.^[^ ^20^ ^]^

**Figure 1:**
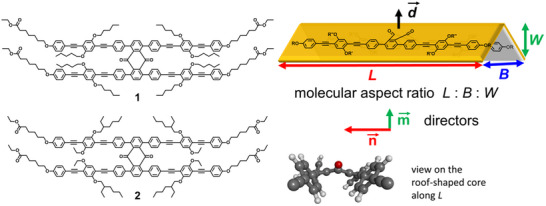
A) Roof‐shaped biaxial lead structure **1**, newly studied mesogen **2**. B) Schematic representation of the mesogen with the definition of the molecular aspect ratio, the major and minor director $\vec{n}$ and $\vec{m}$ and the dipole of the anthraquinone core.

In an attempt to tailor nematogens with optimum molecular biaxiality, we designed the roof‐shaped molecule **2** as a derivative from the lead structure **1** synthesised to realise biaxial nematic mesophases (**Figure** **1**).^[^ ^21^ ^]^ Compared to **1** we shortened one set of the lateral alkoxy chains and increased the volume of the second set by using the 2‐ethylhexyl group in mesogen **2**, which should keep the bulkier alkoxy chains pointing to the periphery of the molecule and thus the structure should be conformationally more defined along the minor axis B. As shown earlier by Norvez et al. the 1,4,5,8‐tetrasubstituted anthraquinone generates a bent structure with a large dipole moment of up to 4.9 Debye along the apex, which is expected to be favorable for the alignment of the second minor molecular axis (W).^[^ ^22^ ^]^ XRS studies and NMR relaxometry of **1** indeed confirmed biaxial nematic domains but a macroscopic uniform alignment for the formal, convincing proof of this outstanding and elusive phase is still lacking. However, if our structural hypothesis for the nematic phases is valid and the nematic phases are not only aligned along the principal director $\vec{n}$ but also along the minor director $\vec{m}$, i.e. the rotation about the long axis is hindered, in the nematic the conjugated units should always be in contact and thus the structure will be favorable for charge migration.

Thermotropic biaxial low‐molecular mass nematogens were the subject of extensive research over more than 30 years, after the formation of a biaxial nematic mesophase has been predicted by Freiser.^[^ ^23^ ^]^ Board and bent‐shaped mesogens were prepared, but eventually, no material have been confirmed to exhibit a biaxial nematic phase.^[^ ^24^ ^]^ For board‐shaped mesogens maximum molecular biaxiality has been proposed by Straley^[^ ^25^ ^]^ and by Luckhurst^[^ ^26^ ^]^ with an aspect ratio length ***L***: breath ***B***: width W to be 10: 3.16: 1 or 15: 5: 3, respectively. Theory predicted that with such molecules, the probability of finding a biaxial nematic mesophase will be highest.

Herein, we demonstrate the synthesis and structural study of target nematogen **2** with an aspect ratio close to the maximum of molecular biaxiality according to Straley. It shows a nematic phase over a broad temperature range larger than 100 K. The nematic material aligns homeotropically between the glass substrates and planar in the liquid crystal cells. Comprehensive TOF studies revealed that these materials are exceptional, showing exclusively electron conduction with a record charge carrier mobility approaching 2.2 × 10^−2^ cm^2 ^V^−1 ^s^−1^ for low molar mass nematic materials. Molecular dynamics simulations of **1** indicate that these observations have their origin in an emerging biaxial ordering in the nematic phase (Figure S16, Supporting Information).

## 2 Results and Discussion

The target nematogen **2** was synthesised by an optimised literature procedure (see ESI).^[^ ^21^ ^]^ The final cross‐coupling step between a *tetra*‐4‐iodophenylanthraquinone core and an arm with a terminal alkyne group yielded the product in a fourfold Sonogashira reaction. After a thorough purification by column chromatography and subsequent recycling‐GPC, 86% of the LC material was isolated, corresponding to a yield of 96% per individual coupling reaction. The identity and purity were confirmed by the GPC elugram, NMR spectroscopy techniques, high resolution mass spectrometry, and elemental analysis (ESI).

**Figure 2:**
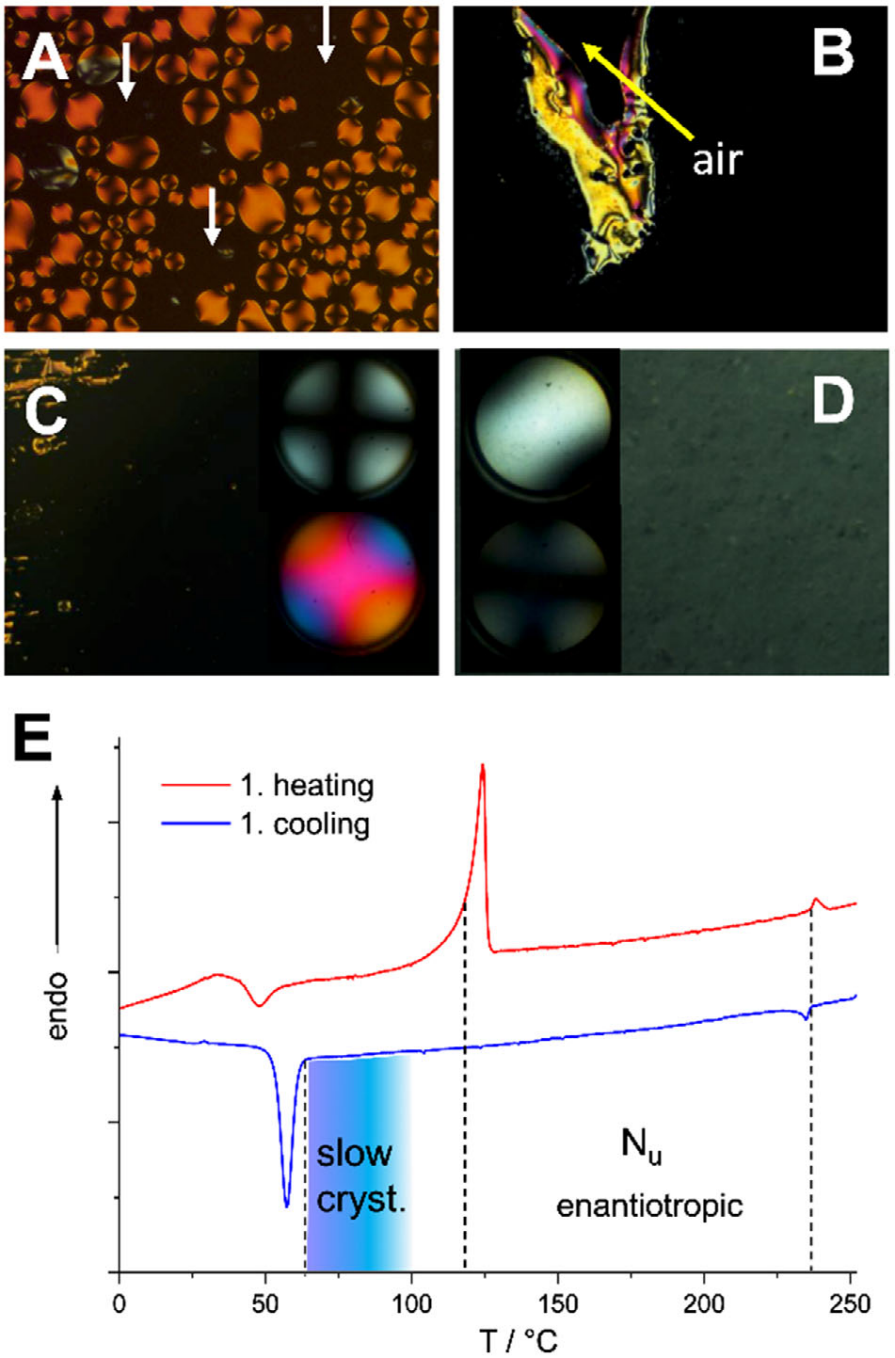
Thermotropic properties of compound **2**. Orthoscopy and Conoscopy. A) Nematic droplets transforming to the homeotropic phase at 235.2 °C. B) Homeotropic aligned nematic phase at 233.5 °C and the nematic Schlieren texture formed around an air bubble. C) Homeotropic texture at 107 °C and the corresponding conoscopic images as insets without and with λ compensator showing a uniaxial, optical positive phase. D) Uniform birefringent texture after quenching to room temperature and annealing at 102.4 °C, and the corresponding conoscopy images (insets) at 0° and rotated by 45°. E) DSC curves at a heating/cooling rate of 10 K min^−1^.

Differential scanning calorimetry confirms the clearing transition with a low hysteresis (0.5 K) and small transition enthalpy (1.7 kJ mol^−1^), which agrees with a first order I‐N transition (**Figure** **2**E, **Table** **1**). A second phase transition from a crystalline to a liquid crystal phase is observed at low temperature with a rather large hysteresis of 58 K and a substantial transition enthalpy (55 kJ mol^−1^). The temperature range of the stable enantiotropic liquid crystal phase is broad and exceeds 100 K.

**Table 1:** Thermotropic properties of **2** (heating rate 10 K min^−1^).

	Transition Temperatures (Onset) in °C / Enthalpies in kJ mol^−1^
1. Heating^a)^	Cr 118.9 / 55.3 N 236.6 / 1.7 I
2. Cooling	I 236.1 / ‐1.7 N 60.9 / ‐34.7 Cr

^a)^ Cr crystal, N nematic liquid crystal, I isotropic liquid.

Polarised optical microscopy (POM) of **2** sandwiched between two glass slides exhibits nematic droplets upon cooling from the isotropic phase, which change to a homeotropically aligned phase at 235 °C (Figure 2). Around air bubbles, the homeotropic texture transformed to a Schlieren texture reminiscent for a nematic self‐assembly of the mesogens. A conoscopic study of the homeotropic areas revealed a uniaxial optical positive phase at 220 °C, which did not change upon slow cooling to 107 °C. However, fast cooling or fast heating generated a uniform birefringence and conoscopy exhibited a strong splitting of the isogyres, which slowly relaxed to the uniaxial phase (Figure 2D). Earlier studies on the homologous compound **1** by NMR relaxometry demonstrated that this type of mesogens self‐assemble into small biaxial aggregates.^[^ ^21^ ^]^ In the homeotropically oriented nematic phase of **2**, such domains are obviously not macroscopically aligned along the minor director, resulting in an uniaxial cross in conoscopy. The present results suggest that a weak and short‐lived macroscopic biaxiality can be induced through rapid thermal transitions or mechanical stress. However, this alignment is not sustained, and we do not claim the presence of a stabilized biaxial nematic phase. The nematic phase can be supercooled and starts to crystallise only slowly below 100 °C. In the time scale of the present experiments, no crystallisation is observed upon cooling to 100 °C. It is also important to note that heating the material to 250 °C results in a slow decomposition (Figure S12, Supporting Information second heating cycle). Therefore, all samples and devices have been prepared at 170 °C or lower temperatures to exclude degradation.

**Figure 3:**
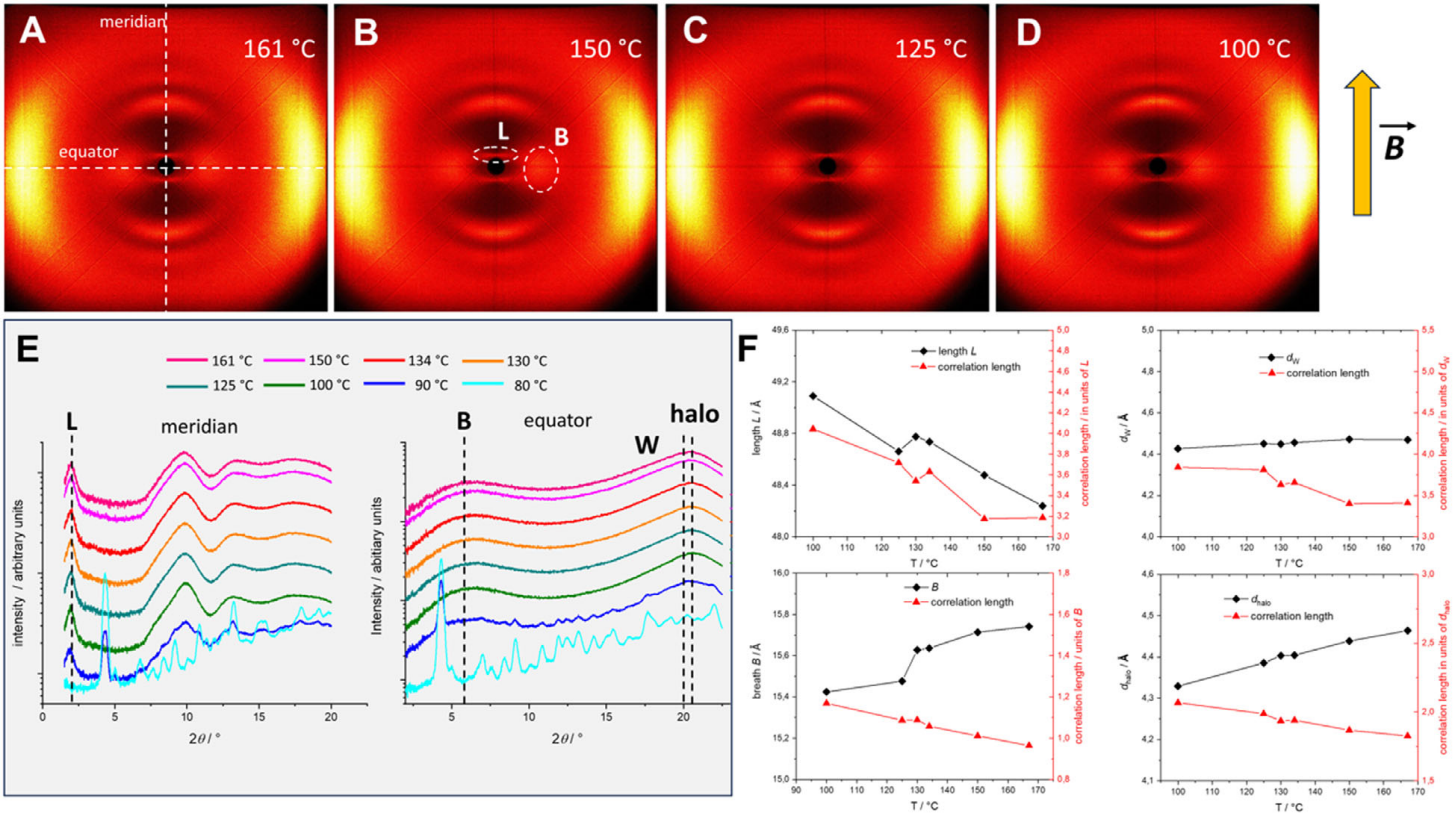
Temperature‐dependent XRS studies of compound **2** in the magnetic field parallel to the meridian of the pattern. A–D) Wide‐angle patterns at different temperatures in the nematic phase. E) Intensity profiles along the meridian and the equator. F) X‐ray parameters *L*, *B*, stacking distance *d_W_* along *W*, and average distance *d*_halo_ between hydrocarbons (black lines) and the corresponding correlation lengths (red lines).

XRS studies of a magnetic field‐aligned (1 T) material confirm these results (**Figure** **3**). The XRS patterns taken in the nematic phase reveal only broad diffuse scattering signals typical for anisotropic fluids, in which molecules possess only next‐neighbour correlations. The molecules align with their long axis along the meridian owing to the diamagnetic anisotropy of the aromatic units. The small angle signal can be attributed to the molecular length *L*. All further broad meridional scattering maxima are rationalised by the highly symmetric molecular scaffold, producing further periodicity along the principal axis.^[^ ^21^ ^]^ This special pattern is also confirmed by XRS simulation of a model of the lead structure **1** energy minimised by molecular dynamics calculations (see ESI for details and Figures S16 and S17, Supporting Information). Along the equator two broad signals are evident, one for the molecular breadth *B* and one for the aliphatic chain distance *d*_halo_ superimposed with that for the stacking along the aromatic faces W (*d_W_*) (see Figures S14 and S15, Supporting Information). Because of the roof‐shape of the mesogen (Figure 1) *d_W_* is not equal to the total width *W* and therefore *W* is not easily accessible. However, it can be estimated via the density which is determined to be 0.92 g/cm^3^ at 100 °C (see ESI). Knowing *L* (49.1 Å) and *B* (15.4 Å), *W* can be calculated and found to be 6.3 Å at 100 °C (see ESI for details). The aspect ratio L:B:W is consequently 10.06: 3.16: 1.29 and is therefore close to the maximum molecular biaxiality of 10.00: 3.16: 1.00 claimed by Straley.^[^ ^25^ ^]^ The over‐all XRS pattern is dominated by the form factor of the molecules and is reminiscent of a rectangular shape.^[^ ^21^, ^27^ ^]^

X‐ray scattering confirms that upon slow cooling *L* becomes larger, *B* smaller, the distances between hydrocarbons change only slightly and the correlation lengths increase in all cases but remain still in the range of molecular liquids (Figure 3F). These parameters are all favorable for the observation of a biaxial nematic phase, nevertheless, the slow cooling did obviously not result in the permanent macroscopic alignment of the minor axes confirmed by POM observation. Consequently, the absence of a large mono domain prevents the direct proof of phase biaxiality.

**Figure 4:**
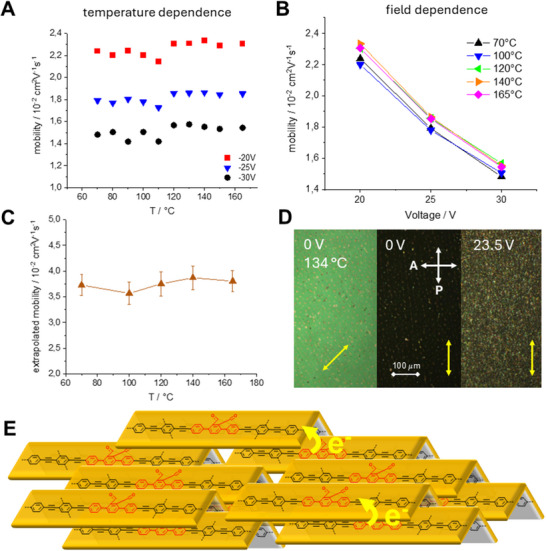
Time‐of‐Flight (TOF) studies in a LC cell with a planar alignment layer and a cell thickness of 8.7 µm (details see ESI). A) Temperature‐dependent electron mobility at three different voltages. B) *E*‐field dependence of the electron mobility. C) The brown curve represents the virtual mobility extrapolated to zero *E*‐field. D) POM images of the cell at 134 °C at 0 V and at 23.5 V. Left and middle texture at 0 V: planar aligned material at 45° and 0° (yellow arrows) with respect to the polarisers (P polariser, A analyser). Right: Texture after the application of 23.5 V. E) Schematic model of the local biaxial order promoting electron mobility. Flexible chains are omitted for clarity.

Although the conjugated parts of the molecules are shielded via an aliphatic shell along *L* and *B*, XRS and former NMR relaxometry results indicate that there must be stacking within the aromatic parts of this family of nematogens. This implies that for planar alignment of nematogens **2** between two electrodes, this nematic material can exhibit enhanced charge carrier transport properties. These considerations prompted us to study the nematogens by time‐of‐flight charge carrier mobility studies (TOF) in LC cells with planar alignment layers between two ITO electrodes upon cooling (**Figure** **4**). It is important to note that a photocurrent signal was observed up to 165 °C but diminished at higher temperatures. The material started to exhibit photocurrents at a voltage of −20 V. The temperature‐dependent study reveals the highest mobility of 0.023 cm^2^V^−1^s^−1^ between 120 and 150 °C, which slightly decreases at lower temperature, most probably owing to very slow crystallisation. The transported charges are exclusively electrons. The n‐type transport can be explained by the electron‐deficient nature of the anthraquinone core.

Additionally, a strong anomalous negative field‐dependence of the mobility is uncovered (Panel B). Extrapolation of the mobility to zero field furnishes even higher mobilities in the range of 0.036–0.039 cm^2^V^−1^s^−1^ (Panel C). In principle, this behaviour may be explained by the theory of Bässler et al., which shows that in disordered solids, non‐parallel charge conduction pathways with respect to the electric field are favored at low field strengths because of optimised structural conditions, while the parallel pathway is disfavored because of deep traps.^[^ ^28^ ^]^ When the field increases, the driving force to take the slower parallel pathway is increasing, other pathways are less probable or excluded, and the mobility drops. However, the present material is uniform in a highly fluid state of matter and the nematogens contain a core which have been found to realise a high dipole moment. This implies that upon application of an external field the dipoles may align opposite to it, and the local field enforcing charge transport decreases. The LC cells were consequently studied with POM and second harmonic generation to record the response to the applied electronic field. The POM study of the LC cells is performed at 0 V and −23.5 V. The dark texture at 0 V changes to be uniformly bright when rotated by 45° confirming the planar alignment of the material. At −23.5 V a strongly birefringent texture appears, which does not change upon rotation of the sample. This can best be explained by electroconvection.^[^ ^29^ ^]^ In our measurements, the electroconvection onset was observed at voltages above ≈20.5 V, while some of the TOF experiments were conducted at 20.0 V. Thus, these measured mobilities at 20.0 V are not influenced by convective flow. Furthermore, early experiments suggest that convection‐related flow velocities are several orders of magnitude slower than carrier drift velocities, indicating minimal dynamic interference.^[^ ^30^ ^]^ A preliminary second harmonic generation (SHG) study, exhibited weak SHG activity pointing to a weak polar order of the material (see Figure S19, Supporting Information). While the electroconvection produces disorder, the applied electric field aligns the dipoles to give polar clusters. However, electroconvection – which emerges only at higher voltages–may contribute to dynamic disorder and domain fluctuations. Measurements under smaller fields indicate the intrinsic transport behaviour is unaffected by these instabilities. Consequently, the strong anomalous negative field‐dependence of the charge mobility has presumably two origins: alignment of clusters leading to the reduction of the local field and increasing disorder as a consequence of the electroconvection.

## 3 Conclusion

This is the first nematic material with an almost optimum molecular biaxiality, showing record electron mobility for an anisotropic liquid phase. We propose that the high mobility arises from local, transient biaxial domains in which aromatic units are favorably stacked, even though macroscopic biaxial order is not achieved. Thus, molecules are aligned along the major and the two minor axes. This situation exists only in small domains and not over the whole sample, i.e., why a macroscopic persistent biaxial nematic phase cannot be confirmed.

The authors have cited additional references within the Supporting Information.^[^ ^31^, ^32^, ^33^, ^34^, ^35^, ^36^, ^37^, ^38^, ^39^, ^40^, ^41^, ^42^, ^43^, ^44^, ^45^, ^46^, ^47^, ^48^, ^49^, ^50^, ^51^, ^52^, ^53^, ^54^, ^55^, ^56^, ^57^, ^58^, ^59^, ^60^ ^]^

## Conflict of Interest

The authors declare no conflict of interest.

## Supporting information



Supporting Information

## Data Availability

The data that support the findings of this study are available in the supplementary material of this article.
