# Supplementary material for: High Electron Charge Carrier Mobility in the Nematic Phase of a Roof‐Shaped Nematogen with Optimum Molecular Biaxiality
Source: Adv Sci (Weinh). 2025 Jul 25;12(38):e10009. doi: 10.1002/advs.202510009 (PMC12520507; doi:10.1002/advs.202510009)
Supplement: Supplementary file 1 — Supporting Information [file ADVS-12-e10009-s001.pdf]

ADVANCED SCIENCE
Open Access

Supporting Information

for *Adv. Sci.*, DOI 10.1002/advs.202510009

High Electron Charge Carrier Mobility in the Nematic Phase of a Roof-Shaped Nematogen with Optimum Molecular Biaxiality

Matthias Lehmann, Nikolai Scheuring, Loïc Mager, Dharmendra Pratap Singh*, Richard Mandle* and Alexey Eremin*

Electronic Supplementary Materials

High electron charge carrier mobility in the nematic phase of a roof-shaped nematogen with optimum molecular biaxiality

Matthias Lehmann, Nikolai Scheuring, Loïc Mager, Dharmendra Pratap Singh, Richard Mandle and Alexey Eremin

Content

1 Equipment 3

2 Chemicals 4

3 Synthesis 5

3.1 Synthesis of the core 7

Synthesis of (4-(Trimethylsilyl)phenyl)boronic acid **5** 7

1,4,5,8-Tetrakis(4-(trimethylsilyl)phenyl)anthracene-9,10-dione **7** 9

1,4,5,8-Tetrakis(4-iodophenyl)anthracene-9,10-dione **8** 10

3.2 Synthesis of the arm 11

4-Ethoxyphenyl acetate **12** 12

3-Bromo-4-ethoxyphenyl acetate **13** 13

3-Bromo-4-ethoxyphenol **14** 14

2-Bromo-1-ethoxy-4-((2-ethylhexyl)oxy)benzene **15** 15

1-Bromo-2-ethoxy-5-((2-ethylhexyl)oxy)-4-iodobenzene **16** 16

((5-Ethoxy-2-((2-ethylhexyl)oxy)-4-(trimethylsilyl)ethynyl)phenyl)ethynyl)triisopropyl-silane **17** 17

((5-Ethoxy-2-((2-ethylhexyl)oxy)-4-ethynylphenyl)ethynyl)triisopropylsilane **18** 18

Ethyl 6-(4-((2-ethoxy-5-((2-ethylhexyl)oxy)-4-((triisopropylsilyl)ethynyl)phenyl)-ethyn-yl)-phenoxy)hexanoate **19** 19

Ethyl 6-(4-((2-ethoxy-5-((2-ethylhexyl)oxy)-4-ethynylphenyl)ethynyl)phenoxy)-hexan-oate **20** 20

3.3 Synthesis of Nematogen **2** 21

4 NMR, IR, Mass data and GPC Elugram 23

5 Sample preparation 26

6 DSC studies 27

7 X-ray scattering 28

8 Density Estimation and Calculation of the Width *W* 30

9 DFT calculation, MD Simulation and XRS simulation 31

10 Time-of-flight (TOF) charge carrier mobility 34

11 References 35

1 Equipment

Preparative recycling gel permeation chromatography (GPC). GPC was performed with the liquid chromatograph *LC-20A* (Shimadzu). The column set (*PSS SDV 50 Å*, 20·600 mm; *PSS SDV 500 Å*, 20·600 mm) was eluted with HPLC-grade $CHCl_3$ at a flow rate of 4.0 $mL \cdot min^{-1}$.

Nuclear magnetic resonance spectroscopy (NMR). NMR spectra were recorded on a Bruker-Daltonics *Avance-400* spectrometer operating at 400 MHz (^1H) or 100 MHz (^{13}C), with the residual solvent signal used as the internal standard.

MALDI mass spectrometry. Mass spectra were recorded on a Bruker-Daltonics *autflex II* (MALDI) and on a Bruker-Daltonics *ultrafleXtreme* (HRMS-MALDI).

Polarized optical microscopy (POM). The studies of optical textures of the mesophases were realized with a Nikon Eclipse *LV100Pol* optical polarizing microscope equipped with a Linkam *LTS420* heating stage and a Linkam *T95-HS* system controller. The POM heating system was calibrated by commercially available compounds.

X-ray scattering (XRS). The temperature dependent SAXS and WAXS X-ray investigations were performed on a Bruker *Nanostar* (Detector *Vantec2000*, Microfocus copper anode X-ray tube *Incoatec*). The powder was transferred to Mark capillary, which was sealed and glued into the metal sample holder with magnets. The XRS heating system was calibrated by liquid crystal standard compounds. The XRS data was evaluated by the program datasqueeze using silver behenate as a calibration standard.

Differential scanning calorimetry (DSC). DSC measurements were performed on a PerkinElmer *DSC 8500* equipped with cryofill cooling system and Pt/Ir ovens. The samples were sealed in TA hermetic pans and lids. All measurements were performed with a rate of 10 K/min and were evaluated with the Pyris Software for Windows.

FT-IR microscopy. FT-IR spectra were recorded with a JASCO *FT/IR-4600*. The samples were prepared as thin films.

UV-VIS spectroscopy. UV-VIS spectra was recorded with a JASCO *V-770*. The sample was prepared as thin film.

Time-of-Flight measurement: Nd:YAG pulsed laser was used, equipped with a 355 nm excitation wavelength and a 5 ns pulse width. The displacements of holes and electrons were measured using a digital oscilloscope (Keysight, DSOX3022T). The temperature was controlled by the EUROTHERM 3205 temperature controller with an accuracy of ± 0.1°C.

2 Chemicals

1,4,5,8-Tetrachloroanthraquinone (n/a) and ethyl 6-bromohexanoate (97 %) were purchased from abcr GmbH. Trimethylsilylacetylene (98 %) and 4-ethoxyphenol (99 %) were purchased from Acros Organics. Copper(I) iodide (99.99 %) was purchased from Alfa Aesar. 18-[Crown]-6 (99 %) was purchased from Apollo Scientific. Sodium hydroxide (95 %), hydrochloric acid (37 %), sulfuric acid (98 %) and sodium periodate (n/a) were purchased from Fischer Scientific. Potassium carbonate (99 %), sodium carbonate (99.5 %), sodium hydrogen sulfite (40 %) and potassium iodide (99.5 %) were purchased from Grüssing GmbH. Acetic anhydride (99 %) was purchased from Honeywell. 1-Bromo-2-ethylhexane (n/a) was purchased from Merck KGaA. Sodium sulphate (99 %) and iodine (99.5 %) were purchased from ORG Laborchemie. Triisopropylacetylene (97 %), trimethyl borate (98 %), 1,4-dibromobenzene (98 %), trimethylsilyl chloride (n/a), *n*-butyllithium (n/a), bis(diphenylphosphino)ferrocene)palladium(II) dichloride (99 %), iodine monochloride (95 %), 4-iodophenol, Tetrakis(triphenylphosphine)palladium (0) (99 %) and *N*,*N*,*N*-Tributylbutan-1-aminium fluoride (97 %) were purchased from Sigma Aldrich. All chemicals employed were used as received, without further purification. Tetrahydrofuran (99.5%) and chloroform (99.8%) were purchased from Fischer Scientific. Pyridine (99.5 %) and trimethylamine (99 %) were purchased from Grüssing GmbH. Acetonitrile (99.9 %) was purchased from Honeywell. Ethyl acetate, dichloromethane, cyclohexane (>99 %) and acetone were purchased from Jäkle. Ethanol (99.8%), 1,2-dimethoxyethane (n/a) and chloroform-*d* (99.8%) were purchased from Sigma Aldrich. Methanol (>98.5%) was purchased from VWR. Tetrahydrofuran, chloroform, pyridine, acetonitrile, cyclohexane, ethanol, chloroform-*d*, 1,2-dimethoxyethane and methanol were used as received. All other solvents were distilled and, if necessary, dried via standard procedures before use. As inert gas, dry nitrogen was employed. For column chromatography, *silica gel 60* from Macherey Nagel (70-230 mesh) was used. The reactions were controlled via thin film chromatography using silica gel (aluminium plates) from Merck with fluorescence indicator *F254*.

3 Synthesis

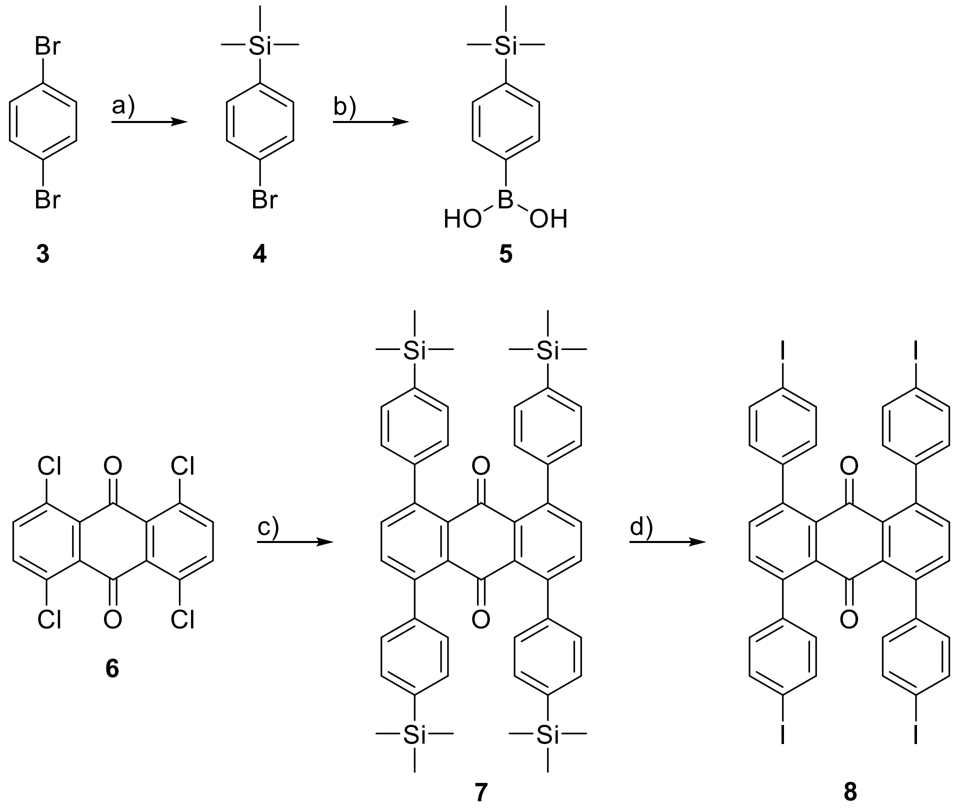

Figure S1. a) 1. 2.5 M n-BuLi, THF, −78 °C, 6 h, 2. TMSCl, RT, 16 h; b) 1. 2.5 M n-BuLi, THF, −78 °C, 0 °C, 1 h, 2. trimethyl borate, −78 °C, RT, 16 h; c) **5**, Pd(dppf)Cl_2, 1,2-Dimethoxyethan:H_2O, 80 °C, 48 h; d) iodine monochloride, $CHCl_3$, RT, 2 h.

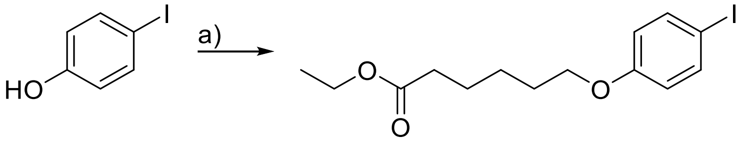

9 **10**

Figure S2. a) Ethyl 6-bromohexanoate K_2CO_3, KI, [18]-Crown-6, acetone, 45 °C, 16 h.

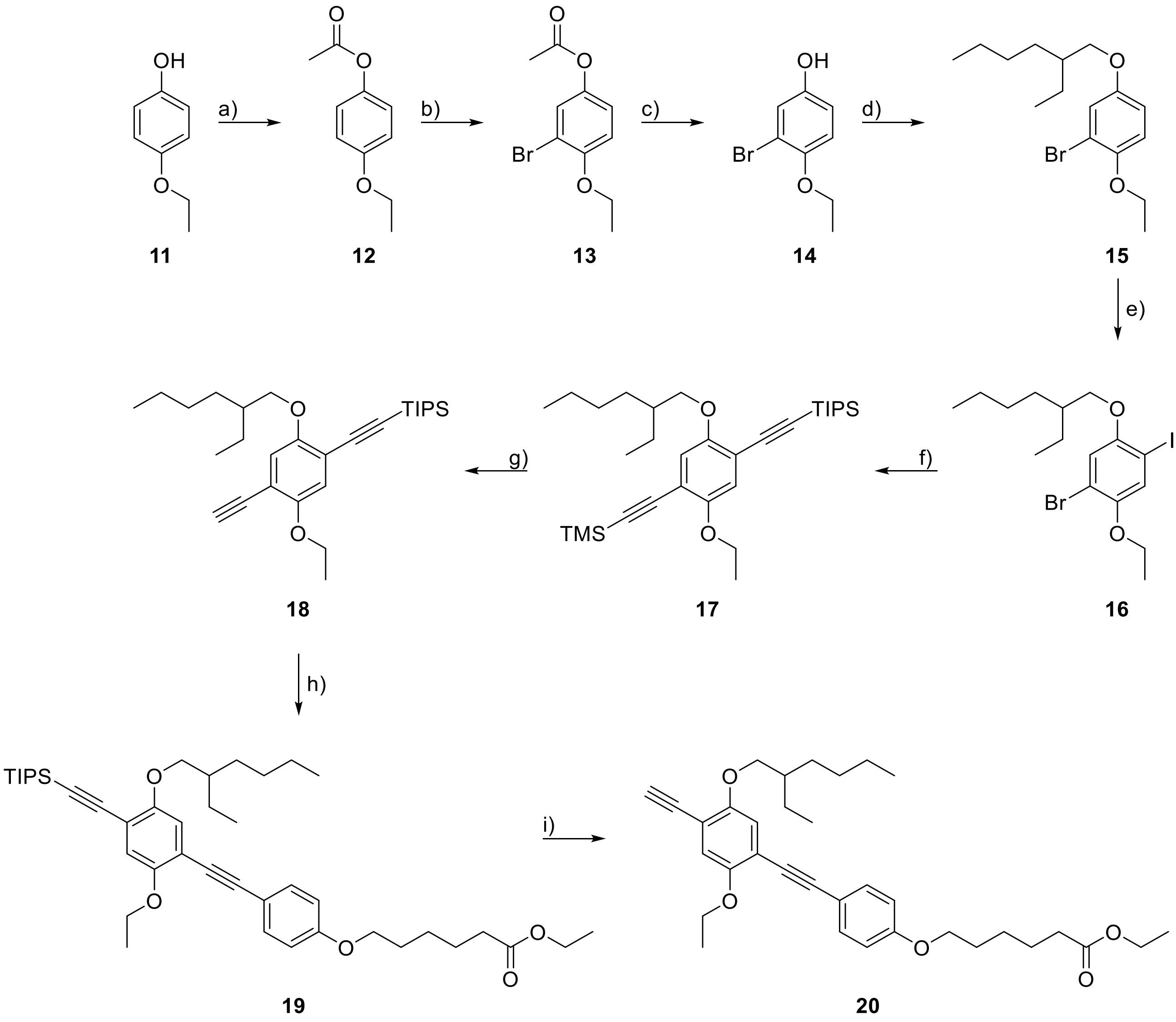

Figure S3. a) Acetic anhydride, Pyridine, 130 °C, 16 h; b) NBS, Acetonitrile, RT–65 °C, 1–3 d; c) 5 M NaOH, methanol, H_2O, RT, 2 h; d) 3-(bromomethyl)heptane, K_2CO_3, KI, [18]-Crown-6, acetone, 65 °C, 16 h; e) I_2, $NaIO_4$, acetic acid, sulfuric acid 50 %, 80 °C, 3 h; f) Pd(dppf)Cl_2, CuI, triethylamine, N_2, 1. TIPSA, 30 °C, 16 h, 2. TMSA, 65 C, 16 h; g) K_2CO_3, methanol, THF, RT, 12 h; h) Pd(dppf)Cl_2, CuI, triethylamine, N_2, **10**, 45 °C, 16 h; i) TBAF, THF, RT, 2 h.

3.1 *Synthesis of the core*

*Synthesis of (4-(Trimethylsilyl)phenyl)boronic acid **5***

(4-Bromophenyl)trimethylsilane ***4***

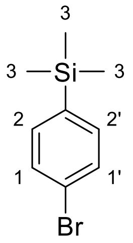

(4-Bromophenyl)trimethylsilane **4** was synthesized according to the literature.[1]

1,4-Dibromobenzene **3** (12.0 g, 50.7 mmol, 1.0 eq.) was dissolved in dry THF (100 mL) and cooled to -78 °C in an inert gas atmosphere. 2.5 M *n*-Butyllithium (3.28 g, 51.2 mmol, 20.5 mL, 1.01 eq.) was added dropwise over 1 h. The solution was stirred for 5 h at -78 °C. Chlorotrimethylsilane (6.05 g, 55.7 mmol, 7.09 mL, 1.1 eq.) was added then and the reaction was stirred for another 16 h at RT.

Distilled water (50 mL) and ethyl acetate (50 mL) were added. The organic phase was washed with distilled water (three times 75 mL) and the aqueous phase was extracted with ethyl acetate (100 mL). The organic phase was dried over sodium sulphate and the solvent was removed under reduced pressure. A colourless oil was received. No further purification was needed.

<u>Yield:</u> 10.2 g (44.5 mmol; 88 %) colourless solid.

^{1}H NMR (400 MHz, $CDCl_3$): δ = 0.26 (s, 9 H, H-3), 7.38 (AA'BB', 2 H, H-2/2'), 7.49 (AA'BB', 2 H, H-1/1') ppm.

NMR data agrees with literature.[1]

(4-(Trimethylsilyl)phenyl)boronic acid **5**

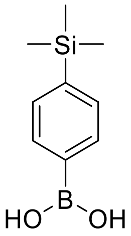

(4-(Trimethylsilyl)phenyl)boronic acid **5** was synthesized according to the literature.[2]

(4-Bromophenyl)trimethylsilane **4** (5.00 g, 21.8 mmol, 1.0 eq.) was dissolved in dry THF (80 mL) and cooled to -78 °C in an inert gas atmosphere. 2.5 M *n*-Butyllithium (1.68 g, 26.2 mmol, 10.5 mL, 1.2 eq.) was added dropwise over 1 h. The solution was stirred for 4 h at -78 °C. Trimethyl borate (3.17 g, 30.5 mmol, 3.45 mL, 1.4 eq.) was added then and the reaction was stirred for another 16 h at RT.

2 M Hydrochloric acid (80 mL) was added and the reaction was stirred for 90 min. The phases were separated and the aqueous phase was extracted with diethyl ether (two times 100 mL). The organic phase was washed with distilled water (three times 75 mL). The organic phase was dried over sodium sulphate and the solvent was removed under reduced pressure. A colourless solid was received. No further purification was needed.

1,4,5,8-Tetrakis(4-(trimethylsilyl)phenyl)anthracene-9,10-dione **7**

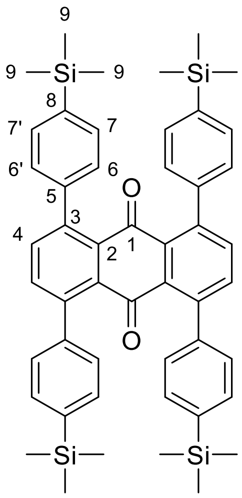

1,4,5,8-Tetrakis(4-(trimethylsilyl)phenyl)anthracene-9,10-dione **7** was synthesized according to the literature.[3]

1,4,5,8-Tetrachloroanthracene-9,10-dione **6** (1.20 g, 3.47 mmol, 1.0 eq.) was dissolved in 1,2-dimethoxyethane:distilled water (105 mL (2:1-mixture (*v*/*v*))) and sodium carbonate (2.94 g, 27.8 mmol, 8.0 eq.). The solution was degassed in nitrogen atmosphere. Pd(dppf)Cl_2 (227 mg, 277 µmol, 0.08 eq.) was added and degassed again. A degassed solution of (4-(Trimethylsilyl)phenyl)boronic acid **3** (4.04 g, 20.8 mmol, 6.0 eq.) in 1,2-dimethoxyethane (15 mL) was added dropwise. The reaction was stirred in an inert gas atmosphere for 48 h at 80 °C. Deionized water (70 mL) was added. The aqueous phase was extracted with ethyl acetate (3 x 80 mL). The organic phase was dried over sodium sulphate and the solvent was removed under reduced pressure. The raw product was purified by column chromatography (silica gel, Cy:DCM = 1:0 to 3:1 (*v*/*v*))

<u>Yield:</u> 1.52 g (1.90 mmol; 55 %) of a yellow solid (mp. >350 °C decomp.).

^{1}H NMR (400 MHz, $CDCl_3$): δ = 0.32 (s, 36 H, H-9), 7.36 (AA'BB', 8 H, H-6/6'), 7.52 (s, 4 H, H-4), 7.53 (AA'BB', 8 H, H-5/5') ppm.

^{13}C NMR (100 MHz, $CDCl_3$): δ = -0.76 (C_p, C-9), 128.24 (C_t, C-6/6'), 133.18 (C_t, C-7/7'), 135.00 (C_q, C-2), 135.53 (C_t, C-4), 139.20 (C_q, C-8), 140.85 (C_q, C-5), 141.68 (C_q, C-3), 187.09 (C_q, C-1) ppm.

FT-IR: $\tilde{\nu}$ = 3060-2895 (s, -C-H, stretching), 1686 (s, -C=O, stretching), 1598 (m, -C=C-, stretching), 1463 (m, -C-H, bending), 1316 (s, -C-H, sym. deformation), 1246, 1206, 1116, 1066, 833 cm^{-1}.

1,4,5,8-Tetrakis(4-iodophenyl)anthracene-9,10-dione **8**

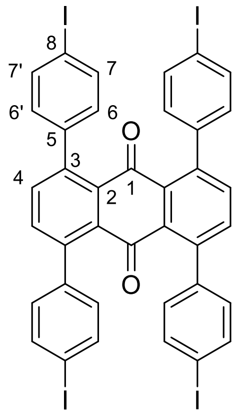

1,4,5,8-Tetrakis(4-iodophenyl)anthracene-9,10-dione **8** was synthesized according to the literature.[4] 1,4,5,8-Tetrakis(4-(trimethylsilyl)phenyl)anthracene-9,10-dione **7** (284 mg, 354 µmol, 1.0 eq.) was dissolved in pre-dried chloroform (75 mL) and degassed in nitrogen atmosphere. Iodine monochloride (284 mg, 354 µmol, 92.0 µL, 5.0 eq.) as 1 M solution in pre-dried chloroform (1.25 mL) was added. The reaction was stirred for 1 h at RT. Sodium disulfite (20 mL) was added. The organic phase was separated and washed with distilled water (two times 100 mL) and dried over sodium sulphate. The solvent was removed under reduced pressure. The raw product was purified by precipitation from chloroform in methanol.

Yield: 307 mg (302 µmol; 85 %) of a yellow solid (mp. >350 °C decomp.).

^{1}H NMR (400 MHz, $CDCl_3$): δ = 6.98 (AA'BB', 8 H, H-6/6'), 7.50 (s, 4 H, H-4), 7.68 (AA'BB', 8 H, H-7/7') ppm.

^{13}C NMR (100 MHz, $CDCl_3$): δ = 93.88 (C_q, C-8), 130.80 (C_t, C-6/6'), 134.63 (C_t, C-4), 135.16 (C_q, C-2), 137.30 (C_t, C-7/7'), 138.95 (C_q, C-5), 140.58 (C_q, C-3), 187.30 (C_q, C-1) ppm.

FT-IR: $\tilde{\nu}$ = 1717 (m, -C=O, stretching), 1673 (m, -C=C-, stretching), 1654 (m, -C=C-, stretching), 1275, 1260, 1004, 763 cm^{-1}.

3.2 *Synthesis of the arm*

Ethyl 6-(4-iodophenoxy)hexanoate **10**

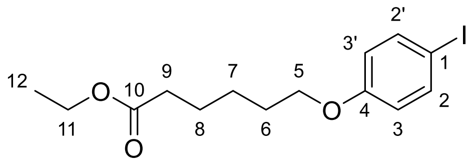

Ethyl 6-(4-iodophenoxy)hexanoate **10** was synthesized according to the literature.[5] 4-Iodophenol **9** (1.35 g, 6.14 mmol, 1.0 eq.) was dissolved in acetone (75 mL) before potassium carbonate (2.54 g, 18.4 mmol, 3.0 eq.) and ethyl 6-bromohexanoate (1.51 g, 6.75 mmol, 1.21 mL, 1.1 eq.) was added. The reaction was stirred for 16 h at 55 °C. After cooling to RT, the colourless solid was filtered off. The solvent was removed under reduced pressure. The residue was dissolved in ethyl acetate (50 mL) and washed with distilled water until pH was neutral. The solvent was removed under reduced pressure. The raw product was purified by column chromatography (silica gel, Cy:EE = 50:1 to 12:1 (*v*/*v*))

<u>Yield:</u> 2.06 g (5.69 mmol; 93 %) colourless liquid.

^{1}H NMR (400 MHz, $CDCl_3$): δ = 1.25 (t, ^3J = 7.1 Hz, 3 H, H-12), 1.45 – 1.53 (m, 2 H, H-6/7/8), 1.65 – 1.73 (m, 2 H, H-6/7/8), 1.75 – 1.82 (m, 2 H, H-6/7/8), 2.33 (t, ^3J = 7.5 Hz, 2 H, H-9), 3.91 (t, ^3J = 6.5 Hz, 2 H, H-5), 4.13 (q, ^3J = 7.1 Hz, 2 H, H-11), 6.66 (AA'BB', 2 H), 7.53 (AA'BB', 2 H) ppm.

NMR data agrees with literature.[5]

FT-IR: $\tilde{\nu}$ = 2937-2866 (s, -C-H, stretching), 1731 (s, -C=O, stretching), 1585 (m, -C=C-, stretching), 1572 (s, -C=C-, stretching), 1486 (m, -C-H, bending), 1472 (m, -C-H, bending), 1372 (s, -C-H, sym. deformation), 1174, 821 cm^{-1}.

*4-Ethoxyphenyl acetate **12***

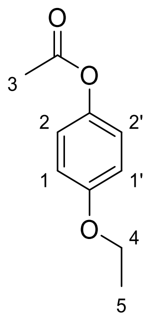

4-Ethoxyphenyl acetate **12** was synthesized according to the literature.[6] 4-Ethoxyphenol **11** (26.3 g, 190 mmol, 1.0 eq.) was dissolved in pyridine (300 mL) and acetic anhydride (25.3 g, 247 mmol, 23.4 mL, 1.3 eq.) was added. The solution was stirred for 3 d at 130 °C. After cooling to RT, the solvent was removed under reduced pressure. The yellow oil was taken up with ethyl acetate (200 mL) and washed with 2 M hydrochloric acid (three times 100 mL). The organic phase was washed with distilled water until pH was neutral. The organic phase was dried over sodium sulphate and the solvent was removed under reduced pressure. The raw product was purified by column chromatography (silica gel, Cy:EE = 5:1 (*v*/*v*)).

Yield: 32.9 g (182 mmol, 96 %) colourless solid (mp. 52.3–54.3 °C).

^{1}H NMR (400 MHz, $CDCl_3$): δ = 1.41 (t, ^3J = 7.0 Hz, 3 H, H-5), 2.27 (s, 3 H, H-3), 4.01 (q, ^3J = 7.0 Hz, 2 H, H-4), 6.87 (AA'BB', 2 H, H-2/2'), 6.99 (AA'BB', 2 H, H-1/1') ppm.

NMR data agrees with literature.[7]

FT-IR: $\tilde{\nu}$ = 2986-2935 (s, -C-H, stretching), 1762 (s, -C=O, stretching), 1746 (s, -C=C-, stretching), 1595 (m, -C=C-, stretching), 1507 (s, -C=C-, stretching), 1473 (m, -C-H, bending), 1374 (s, -C-H, sym. deformation), 1180, 905 cm^{-1}.

*3-Bromo-4-ethoxyphenyl acetate **13***

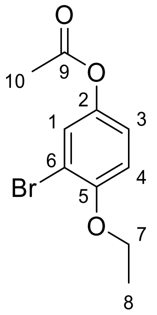

3-Bromo-4-ethoxyphenyl acetate **13** was synthesized according to literature.[8] 4-Ethoxyphenyl acetate **12** (32.9 g, 182 mmol, 1.0 eq.) was dissolved in acetonitrile (400 mL) and NBS (32.5 g, 182 mmol, 1.0 eq.) was added slowly. The reaction was stirred for 3 d at 65 °C. Sodium disulfite (20 %,150 mL) was added. The aqueous phase was extracted with ethyl acetate (three times 200 mL) and the organic phase was washed with distilled water (two times 150 mL) and brine (150 mL). The solvent was dried over sodium sulphate and removed under reduced pressure. No further purification was needed.

Yield: 42.6 g (164 mmol; 90 %) yellowish liquid.

^{1}H NMR (400 MHz, $CDCl_3$): δ = 1.46 (t, ^{3}J = 7.0 Hz, 3 H, H-8), 2.27 (s, 3 H, H-10), 4.08 (q, ^{3}J = 7.0 Hz, 2 H, H-7), 6.86 (d, ^{3}J = 8.9 Hz, 1 H, H-4), 6.99 (dd, ^{3}J = 8.9 Hz, ^{4}J = 2.7 Hz, 1 H, H-3), 7.30 (d, ^{4}J = 2.8 Hz, 1 H, H-1) ppm.

^{13}C NMR (100 MHz, $CDCl_3$): δ = 14.84 (C_p, C-8), 21.11 (C_p, C-10), 65.39 (C_s, C-7), 112.15 (C_q, C-6), 113.28 (C_t, C-4), 121.37 (C_t, C-3), 126.64 (C_t, C-1), 144.12 (C_q, C-2), 153.44 (C_q, C-5), 169.67 (C_q, C-9) ppm.

FT-IR: $\tilde{\nu}$ = 2980-2888 (s, -C-H, stretching), 1751 (s, -C=O, stretching), 1717 (s, -C=C-, stretching), 1489 (m, -C-H, bending), 1472 (m, -C-H, bending), 1368 (s, -C-H, sym. deformation), 1202, 1180 cm^{-1}.

*3-Bromo-4-ethoxyphenol **14***

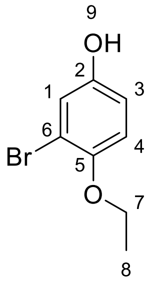

3-Bromo-4-ethoxyphenol **14** was synthesized according to literature.[9] 3-Bromo-4-ethoxyphenyl acetate **13** (41.7 g, 161 mmol, 1.0 eq.) was dissolved in methanol : distilled water (660 mL (7:1-mixture (*v*/*v*))) and 5 M sodium hydroxide (60 mL) solution was added slowly. The reaction was stirred for 2 h at RT before 2.4 M hydrochloric acid was added until pH = 1. Cyclohexane : ethyl acetate (200 mL (1:1-mixture (*v*/*v*))) was added and the aqueous phase was extracted with this mixture (three times 100 mL). The organic phase was washed with distilled water until pH neutral. The solvent was dried over sodium sulphate and removed under reduced pressure. No further purification was needed.

Yield: 14.0 g (64.5 mmol; 40 %) orange-red oil.

^{1}H NMR (400 MHz, $CDCl_3$): δ = 1.43 (t, ^{3}J = 7.0 Hz, 3 H, H-8), 4.03 (q, ^{3}J = 7.0 Hz, 2 H, H-7), 4.72 (bs, 1 H, H-9, O*H*), 6.73 (dd, ^{3}J = 8.8 Hz, ^{4}J = 2.9 Hz, 1 H, H-3), 6.80 (d, ^{3}J = 9.0 Hz, 1 H, H-4), 7.07 (d, ^{4}J = 2.9 Hz, 1 H, H-1) ppm.

NMR data are analogous to the literature.[10]

FT-IR: $\tilde{\nu}$ = 3314 (br, s, -O-H, stretching), 2979-2894 (s, -C-H, stretching), 1607 (m, -C=C-, stretching), 1490 (m, -C-H, bending), 1474 (m, -C-H, bending), 1392 (s, -C-H, sym. deformation), 1200, 1033 cm^{-1}.

2-Bromo-1-ethoxy-4-((2-ethylhexyl)oxy)benzene ***15***

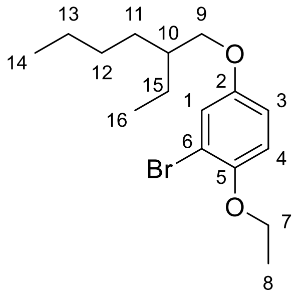

2-Bromo-1-ethoxy-4-((2-ethylhexyl)oxy)benzene **15** was synthesized according to literature.[5] 3-Bromo-4-ethoxyphenol **14** (6.02 g, 27.7 mmol, 1.0 eq.) was dissolved in acetone (300 mL) and potassium carbonate (19.2 g, 139 mmol, 5.0 eq.), potassium iodide (230 mg, 1.39 mmol, 0.05 eq.), [18]-Crown-6 (220 mg, 1832 µmol, 0.03 eq.) and 3-(bromomethyl)heptane (7.56 g, 39.2 mmol, 7.0 mL, 1.4 eq.) were added at RT. The reaction was stirred for 16 h at 55 C and afterwards cooled down to RT. The colourless precipitation was filtered off and the solvent was removed under reduced pressure. The residue was taken up in ethyl acetate and the organic phase was washed with distilled water until pH neutral. The solvent was dried over sodium sulphate and removed under reduced pressure. The raw product was purified by column chromatography (silica gel, Cy:EE = Cy pure to 5:1 (*v*/*v*)).

<u>Yield</u>: 7.10 g (21.6 mmol; 78 %) colourless liquid.

^{1}H NMR (400 MHz, CDCl$_3$): δ = 0.90 (t, ^{3}J = 7.0 Hz, 3 H, H-14), 0.91 (t, ^{3}J = 7.5 Hz, 3 H, H-16), 1.28 – 1.33 (m, 4 H, H-12/13), 1.35 – 1.52 (m, 4 H, H-11/15), 1.43 (t, ^{3}J = 7.0 Hz, 3 H, H-8), 1.64 – 1.73 (m, 1 H, H-10), 3.75 (dd, ^{2}J = 9.0 Hz, ^{3}J = 5.8 Hz, 1 H, H-9A), 3.78 (dd, ^{2}J = 9.0 Hz, ^{3}J = 5.6 Hz, 1 H, H-9B), 4.04 (q, ^{3}J = 7.0 Hz, 2 H, H-7), 6.79 (dd, ^{3}J = 8.9 Hz, ^{4}J = 2.8 Hz, 1 H, H-3), 6.83 (d, ^{3}J = 8.9 Hz, 1 H, H-4), 7.12 (d, ^{4}J = 2.8 Hz, 1 H, H-1) ppm.

^{13}C NMR (100 MHz, CDCl$_3$): δ = 11.24 (C$_p$, C-16), 14.24 (C$_p$, C-14), 15.04 (C$_p$, C-8), 23.19 (C$_s$, C-12/13), 23.95 (C$_s$, C-11/15), 29.20 (C$_s$, C-12/13), 30.61 (C$_s$, C-11/15), 39.53 (C$_t$, C-10), 66.00 (C$_s$, C-7), 71.38 (C$_s$, C-9), 112.97 (C$_q$, C-6), 114.47 (C$_t$, C-3), 115.10 (C$_t$, C-4), 119.59 (C$_t$, C-1), 149.64 (C$_q$, C-5), 154.10 (C$_q$, C-2) ppm.

FT-IR: $\tilde{\nu}$ = 2957-2859 (s, -C-H, stretching), 1603 (m, -C=C-, stretching), 1493 (m, -C-H, bending), 1472 (m, -C-H, bending), 1388 (s, -C-H, sym. deformation), 1205, 1031 cm^{-1}.

1-Bromo-2-ethoxy-5-((2-ethylhexyl)oxy)-4-iodobenzene ***16***

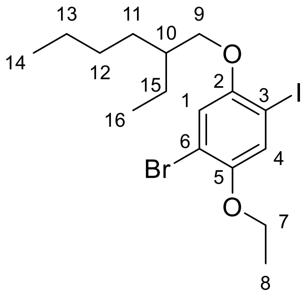

1-Bromo-2-ethoxy-5-((2-ethylhexyl)oxy)-4-iodobenzene **16** was synthesized according to literature.[11] 2-Bromo-1-ethoxy-4-((2-ethylhexyl)oxy)benzene **15** (4.20 g, 12.8 mmol, 1.0 eq.) was dissolved in ethyl acetate:sulfuric acid (50 %) (69 mL (6:1-mixture (*v*/*v*))) and sodium periodate (546 mg, 2.55 mmol, 0.20 eq.) and iodine (1.78 g, 7.02 mmol, 0.55 eq.) were added. The reaction was stirred for 2 h at 80 °C during which time the solution turned into metallic purple. After cooling down to RT, sodium hydrogen sulfite (20 %) was added until no further discoloration was observed. Cyclohexane:ethyl acetate (300 mL (1:1-mixture (*v*/*v*))) was added and the aqueous phase was extracted with this mixture (three times 100 mL). The organic phase was washed with distilled water until pH neutral. The solvent was dried over sodium sulfate and removed under reduced pressure. The raw product was purified by column chromatography (silica gel, Cy:EE = Cy pure to 25:1 (*v*/*v*)).

<u>Yield:</u> 5.04 g (11.1 mmol; 87 %) red liquid.

^{1}H NMR (400 MHz, $CDCl_3$): δ = 0.91 (t, ^{3}J = 7.0 Hz, 3 H, H-14), 0.94 (t, ^{3}J = 7.5 Hz, 3 H, H-16), 1.27-1.37 (m, 4 H, H-12/13), 1.43 (t, ^{3}J = 6.9 Hz, 3 H, H-8), 1.46-1.51 (m, 4 H, H-11/15), 1.69 – 1.78 (m, 1 H, H-10), 3.81 (dd, ^{2}J = 9.1 Hz, ^{3}J = 5.5 Hz, 1 H, H-9A), 3.83 (dd, ^{2}J = 9.7 Hz, ^{3}J = 5.4 Hz, 1 H, H-9B), 4.03 (q, ^{3}J = 7.0 Hz, 2 H, H-7), 6.97 (s, 1 H, H-1), 7.29 (s, 1 H, H-4) ppm.

^{13}C NMR (100 MHz, $CDCl_3$): δ = 11.23 (C_p, C-16), 14.14 (C_p, C-14), 14.82 (C_p, C-8), 23.06 (C_s, C-12/13), 23.95 (C_s, C-11/15), 29.07 (C_s, C-12/13), 30.52 (C_s, C-11/15), 39.43 (C_t, C-10), 66.08 (C_s, C-7), 72.26 (C_s, C-9), 84.57 (C_q, C-3), 112.57 (C_q, C-6), 116.58 (C_t, C-1), 124.55 (C_t, C-4), 150.10 (C_q, C-5), 152.78 (C_q, C-2) ppm.

FT-IR: $\tilde{\nu}$ = 2956-2858 (s, -C-H, stretching), 1743 (s, -C=C-, stretching), 1489 (m, -C-H, bending), 1457 (m, -C-H, bending), 1392 (s, C-H, sym. deformation), 1350, 1207, 1057, 1028 cm^{-1}.

((5-Ethoxy-2-((2-ethylhexyl)oxy)-4-(trimethylsilyl)ethynyl)phenyl)ethynyl)triisopropyl-silane ***17***

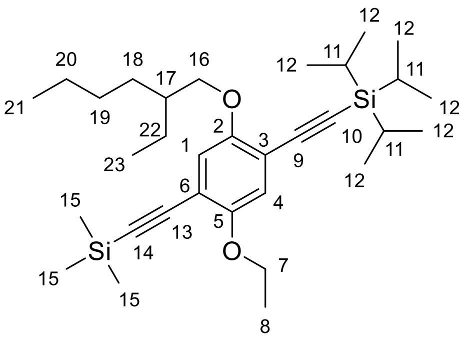

((5-Ethoxy-2-((2-ethylhexyl)oxy)-4-((trimethylsilyl)ethynyl)phenyl)ethynyl)triisopropylsilane **17** was synthesized according to literature.[12] 1-Bromo-2-ethoxy-5-((2-ethylhexyl)oxy)-4-iodobenzene **16** (4.53 g, 9.95 mmol, 1.0 eq.) was dissolved in triethylamine (120 mL) and degassed in nitrogen atmosphere. Pd(dppf)Cl_2 (291 mg, 398 µmol, 0.04 eq.) and copper iodide (56.9 mg, 299 µmol, 0.03 eq.) were added and the solution was degassed in nitrogen atmosphere again. Triisopropylsilyl acetylene (1.87 g, 10.3 mmol, 2.30 mL, 1.03 eq.) was added slowly and the reaction was stirred for 16 h at RT. Afterwards trimethylsilyl acetylene (1.03 g, 10.5 mmol, 1.45 mL, 1.05 eq.) was added and the reaction was stirred for 16 h at 55 °C. The solvent was removed under reduced pressure. The raw product was purified by column chromatography (silica gel, Cy:DCM = 25:1 to 16:1 (*v*/*v*)).

Yield: 4.17 g (7.91 mmol; 80 %) yellowish oil.

^{1}H NMR (400 MHz, $CDCl_3$): δ = 0.26 (s, 9 H, H-15), 0.89 (t, ^{3}J = 6.6 Hz, 3 H, H-21), 0.90 (t, ^{3}J = 7.5 Hz, 3 H, H-23), 1.13 (s, 21 H, H-11/12), 1.25 – 1.32 (m, 4 H, H-19/20), 1.37 – 1.48 (m, 3 H, H-18/22A), 1.42 (t, ^{3}J = 7.0 Hz, 3 H, H-8), 1.53 – 1.62 (m, 1 H, H-22B), 1.66 – 1.75 (m, 1 H, H-17), 3.79 (dd, ^{2}J = 8.8 Hz, ^{3}J = 6.0 Hz, 1 H, H-16), 3.84 (dd, ^{2}J = 8.9 Hz, ^{3}J = 5.2 Hz, 1 H, H-16), 4.03 (q, ^{3}J = 7.0 Hz, 2 H, H-7), 6.87 (s, 1 H, H-1), 6.89 (s, 1 H, H-4) ppm.

^{13}C NMR (100 MHz, $CDCl_3$): δ = 0.12 (C_p, C-15), 11.23 (C_p, C-23), 11.49 (C_t, C-11), 14.27 (C_p, C-21), 14.98 (C_p, C-8), 18.85 (C_p, C-12), 23.22 (C_s, C-19/20), 23.78 (C_s, C-22A/22B), 29.22 (C_s, C-19/20), 30.42 (C_s, C-18), 39.68 (C_t, C-17), 65.69 (C_s, C-7), 71.38 (C_s, C-16), 96.47 (C_q, C-10), 100.06 (C_q, C-14), 101.38 (C_q, C-13), 103.00 (C_q, C-9), 114.07 (C_q, C-6), 114.19 (C_q, C-3), 116.37 (C_t, C-1), 118.63 (C_t, C-4), 153.71 (C_q, C-5), 154.52 (C_q, C-2) ppm.

FT-IR: $\tilde{\nu}$ = 2956-2864 (s, -C-H, stretching), 2151 (m, -C≡C-, stretching), 1496 (m, -C-H, bending), 1465 (m, -C-H, bending), 1382 (s, -C-H, sym. deformation), 1200, 839 cm^{-1}.

((5-Ethoxy-2-((2-ethylhexyl)oxy)-4-ethynylphenyl)ethynyl)triisopropylsilane ***18***

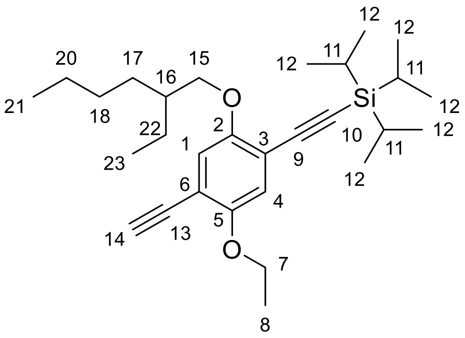

((5-Ethoxy-2-((2-ethylhexyl)oxy)-4-ethynylphenyl)ethynyl)triisopropylsilane **18** was synthesised according to literature.[12-13] ((5-Ethoxy-2-((2-ethylhexyl)oxy)-4-((trimethylsilyl)ethynyl)-phenyl)ethynyl)triisopropylsilane **17** (4.17 g, 7.91 mmol, 1.0 eq.) was dissolved in methanol:THF (120 mL (1:1-mixture (*v*/*v*))). Potassium carbonate (1.53 g, 11.1 mmol, 1.4 eq.) was added and the reaction was stirred for 16 h at RT. Afterwards the potassium carbonate was filtered off and the solvent was removed under reduced pressure. The raw product was purified by column chromatography (silica gel, Cy:DCM = 25:1 to 16:1 (*v*/*v*)).

<u>Yield:</u> 1.65 g (3.63 mmol; 46 %) yellowish oil.

^{1}H NMR (400 MHz, $CDCl_3$): δ = 0.89 (t, ^3J = 7.0 Hz, 3 H, H-20), 0.90 (t, ^3J = 7.5 Hz, 3 H, H-22), 1.13 (s, 21 H, H-11/12), 1.26 – 1.32 (m, 4 H, H-18/19), 1.36 – 1.50 (m, 3 H, H-17/21), 1.43 (t, ^3J = 7.0 Hz, 3 H, H-8), 1.51 – 1.60 (m, 1 H, H-21), 1.66 – 1.75 (m, 1 H, H-16), 3.34 (s, 1 H, H-14), 3.79 (dd, ^2J = 8.8 Hz, ^3J = 5.9 Hz, 1 H, H-15), 3.84 (dd, ^2J = 8.9 Hz, ^3J = 5.2 Hz, 1 H, H-15), 4.07 (q, ^3J = 7.0 Hz, 2 H, H-7), 6.92 (s, 1 H, H-1), 6.92 (s, 1 H, H-4) ppm.

^{13}C NMR (100 MHz, $CDCl_3$): δ = 11.22 (C_p, C-22), 11.48 (C_t, C-11), 14.27 (C_p, C-20), 14.95 (C_p, C-8), 18.84 (C_p, C-12), 23.22 (C_s, C-18/19), 23.76 (C_s, C-21), 29.20 (C_s, C-18/19), 30.42 (C_s, C-17), 39.66 (C_t, C-17), 65.46 (C_s, C-7), 71.47 (C_s, C-15), 80.28 (C_q, C-13), 82.29 (C_t, C-14), 96.67 (C_q, C-10), 102.83 (C_q, C-9), 112.70 (C_q, C-6), 114.61 (C_q, C-3), 116.91 (C_t, C-1), 118.12 (C_t, C-4), 153.66 (C_q, C-5), 154.40 (C_q, C-2) ppm.

FT-IR: $\tilde{\nu}$ = 3314 (m, ≡C-H, stretching), 2924-2863 (s, -C-H, stretching), 2148 (m, -C≡C-, stretching), 1496 (m, -C-H, bending), 1465 (m, -C-H, bending), 1388 (s, -C-H, sym. deformation), 1219, 861 cm^{-1}.

Ethyl 6-(4-((2-ethoxy-5-((2-ethylhexyl)oxy)-4-((triisopropylsilyl)ethynyl)phenyl)-ethynyl)-phenoxy)hexanoate ***19***

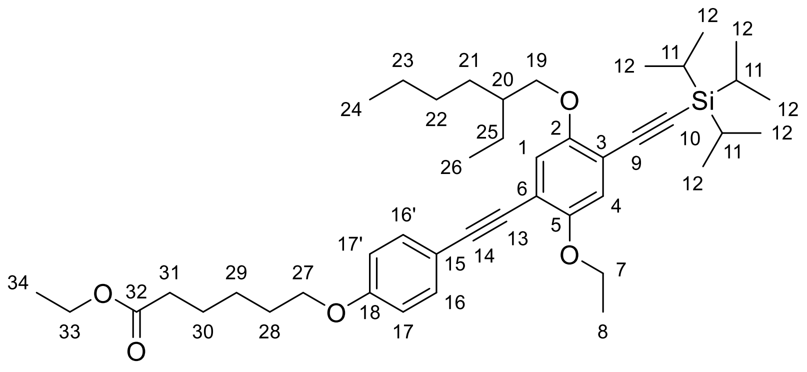

Ethyl 6-(4-((2-ethoxy-5-((2-ethylhexyl)oxy)-4-((triisopropylsilyl)ethynyl)phenyl)ethynyl)-phenoxy)hexanoate **19** was synthesized according to literature.[14] ((5-Ethoxy-2-((2-ethylhexyl)oxy)-4-ethynylphenyl)ethynyl)triisopropylsilane **18** (800 mg, 1.76 mmol, 1.0 eq.) was dissolved in triethylamine (50 mL) and degassed in nitrogen atmosphere. Pd(dppf)Cl_2 (38.6 mg, 52.8 µmol, 0.03 eq.) and copper iodide (16.8 mg, 88.0 µmol, 0.05 eq.) were added and the solution was degassed in nitrogen atmosphere again. Ethyl 6-(4-iodophenoxy)hexanoate (765 g, 2.11 mmol, 1.20 eq.) was added and the reaction was stirred for 16 h at RT. The solvent was removed under reduced pressure. The raw product was purified by column chromatography (silica gel, Cy:DCM = 3:1 to 1:3 (*v*/*v*)).

Yield: 990 mg (1.44 mmol; 82 %) yellowish oil.

^{1}H NMR (400 MHz, $CDCl_3$): δ = 0.90 (t, ^3J = 7.1 Hz, 3 H, H-24), 0.91 (t, ^3J = 7.3 Hz, 3 H, H-26), 1.14 (s, 21 H, H-11/12), 1.26 (t, ^3J = 7.1 Hz, 3 H, H-34), 1.27 – 1.33 (m, 4 H, H-22/23), 1.37 – 1.55 (m, 4 H, H-25/29), 1.46 (t, ^3J = 7.0 Hz, 3 H, H-8), 1.55 – 1.62 (m, 2 H, H-21), 1.67 – 1.76 (m, 3 H, H-20/30), 1.78 – 1.85 (m, 2 H, H-28), 2.34 (t, ^3J = 7.4 Hz, 2 H, H-31), 3.82 (dd, ^2J = 8.9 Hz, ^3J = 5.9 Hz, 1 H, H-19), 3.89 (dd, ^2J = 8.9 Hz, ^3J = 5.2 Hz, 1 H, H-19), 3.97 (t, ^3J = 6.4 Hz, 2 H, H-27), 4.08 (q, ^3J = 6.7 Hz, 2 H, H-7), 4.13 (q, ^3J = 7.1 Hz, 2 H, H-33), 6.85 (AA'BB', 2 H, H-17/17'), 6.94 (s, 2 H, H-1/4), 7.46 (AA'BB', 2 H, H-16/16') ppm.

^{13}C NMR (100 MHz, $CDCl_3$): δ = 11.23 (C_t, C-16), 11.50 (C_t, C-11), 14.27 (C_p, C-24), 14.39 (C_p, C-34), 15.09 (C_p, C-8), 18.85 (C_p, C-12), 23.22 (C_s, C-22/23), 23.78 (C_s, C-25/29), 24.83 (C_s, C-30), 25.76 (C_s, C-21), 29.01 (C_s, C-28), 29.21 (C_s, C-22/23), 30.43 (C_s, C-25/29), 34.36 (C_s, C-31), 39.69 (C_s, C-20), 60.41 (C_s, C-33), 65.66 (C_s, C-7), 67.80 (C_s, C-27), 71.41 (C_s, C-19), 84.81 (C_q, C-9/13), 95.01 (C_q, C-14), 96.16 (C_q, C-10), 103.18 (C_q, C-9/13), 113.44 (C_q, C-3/6), 114.58 (C_t, C-17/17'), 114.73 (C_q, C-3/6), 115.49 (C_q, C-15), 115.96 (C_t, C-1/4), 118.52

(C_t, C-1/4), 133.19 (C_t, C-16/16'), 152.93 (C_q, C-5), 154.67 (C_q, C-2), 159.26 (C_q, C-18), 173.77 (C_q, C-32) ppm.

FT-IR: $\tilde{\nu}$ = 2956-2863 (s, -C-H, stretching), 2148 (m, -C≡C-, stretching), 1734 (s, -C=O, stretching), 1717 (s, -C=C-, stretching), 1511 (s, -C=C-, =C-H, stretching), 1496 (m, -C-H, bending), 1465 (m, -C-H, bending), 1388 (s, -C-H, sym. deformation), 1214, 829 cm^{-1}.

Ethyl 6-(4-((2-ethoxy-5-((2-ethylhexyl)oxy)-4-ethynylphenyl)ethynyl)phenoxy)-hexanoate ***20***

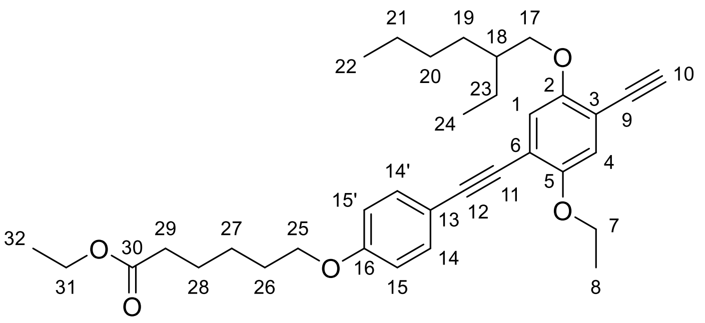

Ethyl 6-(4-((2-ethoxy-5-((2-ethylhexyl)oxy)-4-ethynylphenyl)ethynyl)phenoxy)hexanoate **20** was synthesized according to literature.[13] Ethyl 6-(4-((2-ethoxy-5-((2-ethylhexyl)oxy)-4-((triisopropylsilyl)ethynyl)phenyl)ethynyl)-phenoxy)hexanoate **19** (990 mg, 1.44 mmol, 1.0 eq.) was dissolved in THF (50 mL) and tetrabutylammonium fluoride (544 mg, 1.72 mmol, 1.2 eq.) was added. The reaction was stirred for 3 h at RT before the solvent was removed under reduced pressure. The raw product was purified by column chromatography (silica gel, Cy:DCM = 1:1 to 1:4 (*v*/*v*)).

Yield: 307 mg (576 µmol; 40 %) orange oil.

^{1}H NMR (400 MHz, $CDCl_3$): δ = 0.90 (t, ^3J = 7.0 Hz, 3 H, H-22), 0.93 (t, ^3J = 7.5 Hz, 3 H, H-24), 1.26 (t, ^3J = 7.1 Hz, 3 H, H-32), 1.31 – 1.37 (m, 4 H, H-20/21), 1.44 – 1.55 (m, 6 H, H-19/23/27), 1.46 (t, ^3J = 6.9 Hz, 3 H, H-8), 1.67 – 1.75 (m, 2 H, H-28), 1.77 – 1.85 (m, 3 H, H-18/26), 2.34 (t, ^3J = 7.5 Hz, 2 H, H-29), 3.31 (s, 1 H, H-10), 3.88 (d, ^3J = 5.8 Hz, 2 H, H-17), 3.97 (t, ^3J = 6.3 Hz, 2 H, H-25), 4.07 (q, ^3J = 7.0 Hz, 2 H, H-7), 4.13 (q, ^3J = 7.1 Hz, 2 H, H-31), 6.85 (AA'BB', 2 H, H-15/15'), 6.96 (s, 1 H, H-4), 6.98 (s, 1 H, H-1), 7.46 (AA'BB', 2 H, H-14/14') ppm.

^{13}C NMR (100 MHz, $CDCl_3$): δ = 11.31 (C_p, C-24), 14.25 (C_p, C-22), 14.40 (C_p, C-30), 15.05 (C_p, C-8), 23.19 (C_s, C-20/21), 24.02 (C_s, C-19/23/27), 24.84 (C_s, C-28), 25.77 (C_s, C-

19/23/27), 29.02 (C_s, C-26), 29.19 (C_s, C-20/21), 30.63 (C_s, C-19/23/27), 34.38 (C_s, C-29), 39.49 (C_t, C-18), 60.43 (C_s, C-31), 65.46 (C_s, C-7), 67.83 (C_s, C-25), 72.13 (C_s, C-17), 80.17 (C_q, C-9), 82.20 (C_t, C-10), 84.49 (C_q, C-11), 95.26 (C_q, C-12), 112.21 (C_q, C-3), 114.61 (C_t, C-15/15'), 115.25 (C_q, C-6), 115.39 (C_q, C-13), 116.73 (C_t, C-1), 118.13 (C_t, C-4), 133.22 (C_t, C-14/14'), 153.06 (C_q, C-5), 154.74 (C_q, C-2), 159.33 (C_q, C-16), 173.78 (C_q, C-30) ppm.

MALDI-HRMS: m/z: calculated 532.3183 $[M]^+$, found: 532.3192 $[M]^+$

FT-IR: $\tilde{\nu}$ = 3281 (m, ≡C-H, stretching), 2954-2871 (s, -C-H, stretching), 2208 (w, -C≡C-, stretching), 1734 (s, -C=O, stretching), 1700 (s, -C=C-, stretching), 1512 (s, -C=C-, =C-H, stretching), 1496 (m, -C-H, bending), 1472 (m, -C-H, bending), 1394 (s, -C-H, sym. deformation), 1217, 829 cm^{-1}.

*3.3 Synthesis of Nematogen **2***

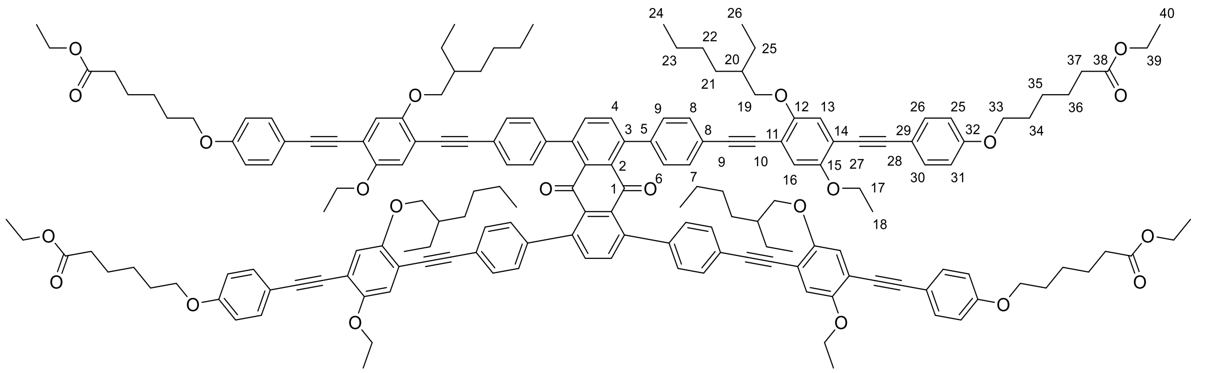

Tetraethyl 6,6',6'',6'''-(((((((9,10-dioxo-9,10-dihydroanthracene-1,4,5,8-tetrayl)tetrakis-(benzene-4,1-diyl))tetrakis(ethyne-2,1-diyl))tetrakis(2-ethoxy-5-((2-ethylhexyl)oxy)benzene-4,1-diyl))tetrakis(ethyne-2,1-diyl))tetrakis(benzene-4,1-diyl))tetrakis(oxy))tetrahexanoate **2** was synthesized according to literature.[14] 1,4,5,8-Tetrakis(4-iodophenyl)anthracene-9,10-dione **8** (40.0 mg, 39.4 µmol, 1.0 eq.) was dissolved in triethylamine : DCM (35 mL (1:1-mixture (*v*/*v*))) and degassed. $Pd(PPh_3)_4$ (18.2 mg, 15.8 µmol, 0.40 eq.) and copper iodide (750 µg, 3.94 µmol, 0.10 eq.) were added and the solution was degassed and kept under nitrogen atmosphere again. The solution was heated up to 45 °C and a separately prepared, degassed solution of ethyl 6-(4-((2-ethoxy-5-((2-ethylhexyl)oxy)-4-ethynylphenyl)ethynyl)phenoxy) **20** (78.2 mg, 155 µmol, 4.5 eq.) in triethylamine : DCM (10 mL (1:1-mixture (*v*/*v*))) was slowly added over 6 h. The reaction was then stirred for further 48 h at 45 °C. The solvent was removed under reduced pressure. The raw product was pre-purified by column

chromatography (silica gel, Cy:EE = 8:1 to 1:1 (*v*/*v*)). The resulting raw product was comprehensively purified by recycling GPC (solvent $CHCl_3$).

<u>Yield</u>: 89.0 mg (33.8 µmol, 86 %) yellow solid.

^{1}H NMR (400 MHz, $CDCl_3$): δ = 0.87 (t, ^3J = 7.1 Hz, 12 H, H-24), 0.96 (t, ^3J = 7.5 Hz, 12 H, H-26), 1.26 (t, ^3J = 7.3 Hz, 12 H, H-40), 1.30 – 1.37 (m, 16 H, H-22/23), 1.43 (t, ^3J = 7.0 Hz, 12 H, H-18), 1.40 – 1.45 (m, 4 H, H-21), 1.47 – 1.56 (m, 16 H, H-21/25/35), 1.58 – 1.66 (m, 4 H, H-25), 1.68 – 1.76 (m, 8 H, H-36), 1.78 – 1.85 (m, 12 H, H-20/34), 2.35 (t, ^3J = 7.5 Hz, 8 H, H-37), 3.86 (dd, ^2J = 9.1 Hz, ^3J = 6.2 Hz, 4 H, H-19), 3.90 (dd, ^2J = 9.0 Hz, ^3J = 5.7 Hz, 4 H, H-19), 3.97 (t, ^3J = 6.4 Hz, 8 H, H-33), 3.98 (q, ^3J = 7.1 Hz, 8 H, H-17), 4.14 (q, ^3J = 7.1 Hz, 8 H, H-39), 6.83 (AA'BB', 8 H, H-31/31'), 6.97 (s, 4 H, H-16), 6.98 (s, 4 H, H-13), 7.30 (AA'BB', 8 H, H-6/6'), 7.46 (AA'BB', 8 H, H-30/30'), 7.52 (AA'BB', 8 H, H-7/7'), 7.54 (s, 4 H, H-4) ppm.

^{13}C NMR (100 MHz, $CDCl_3$): δ = 11.45 (C_p, C-26), 14.29 (C_p, C-24), 14.41 (C_p, C-40), 15.07 (C_p, C-18), 23.25 (C_s, C-22/23), 24.14 (C_s, C-25), 24.85 (C_s, C-36), 25.78 (C_s, C-21/25/35), 29.05 (C_s, C-34), 29.33 (C_s, C-22/23), 30.78 (C_s, C-21), 34.38 (C_s, C-37), 39.70 (C_t, C-20), 60.43 (C_s, C-39), 65.35 (C_s, C-17), 67.81 (C_s, C-33), 72.08 (C_s, C-19), 84.88 (C_q, C-27), 87.00 (C_q, C-10), 94.68 (C_q, C-9), 95.13 (C_q, C-), 113.61 (C_q, C-11), 114.57 (C_t, C-31/31'), 114.59 (C_q, C-14), 115.62 (C_q, C-29), 116.61 (C_t, C-13), 117.12 (C_t, C-16), 123.12 (C_q, C-8), 128.99 (C_t, C-6/6'), 131.33 (C_t, C-7/7'), 133.23 (C_t, C-30/30'), 134.88 (C_q, C-2), 135.34 (C_t, C-4), 139.91 (C_q, C-5), 141.29 (C_q, C-3), 153.28 (C_q, C-15), 154.14 (C_q, C-12), 159.21 (C_q, C-32), 173.78 (C_q, C-38), 186.88 (C_q, C-1) ppm.

MALDI-HRMS: m/z: calculated 2633.3900 $[M]^+$, found: 2633.3888 $[M]^+$

EA: calculated C: 79.30, H: 7.34, found C: 79.17, H: 7.02

Solid state UV-VIS: $\lambda_{max}(\varepsilon)$ = 385 nm, 319 nm

FT-IR: $\tilde{\nu}$ = 2926-2869 (s, -C-H, stretching), 2204 (w, -C≡C-, stretching), 1730 (s, -C=O, stretching), 1683 (s, -C=C-, stretching), 1604 (m, -C=C-, stretching), 1512 (s, -C=C-, =C-H, stretching), 1494 (m, -C-H, bending), 1465 (m, -C-H, bending), 1391 (s, -C-H, sym. deformation), 1213, 826 cm^{-1}.

4 NMR, IR, Mass data and GPC Elugram

Molecule **20**

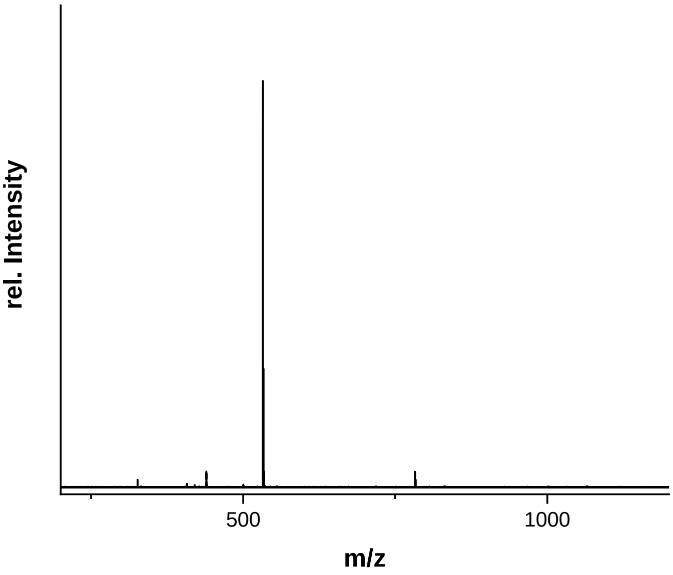

Figure S4. Mass spectra of **20**. Matrix: DCTB; Positive voltage polarity.

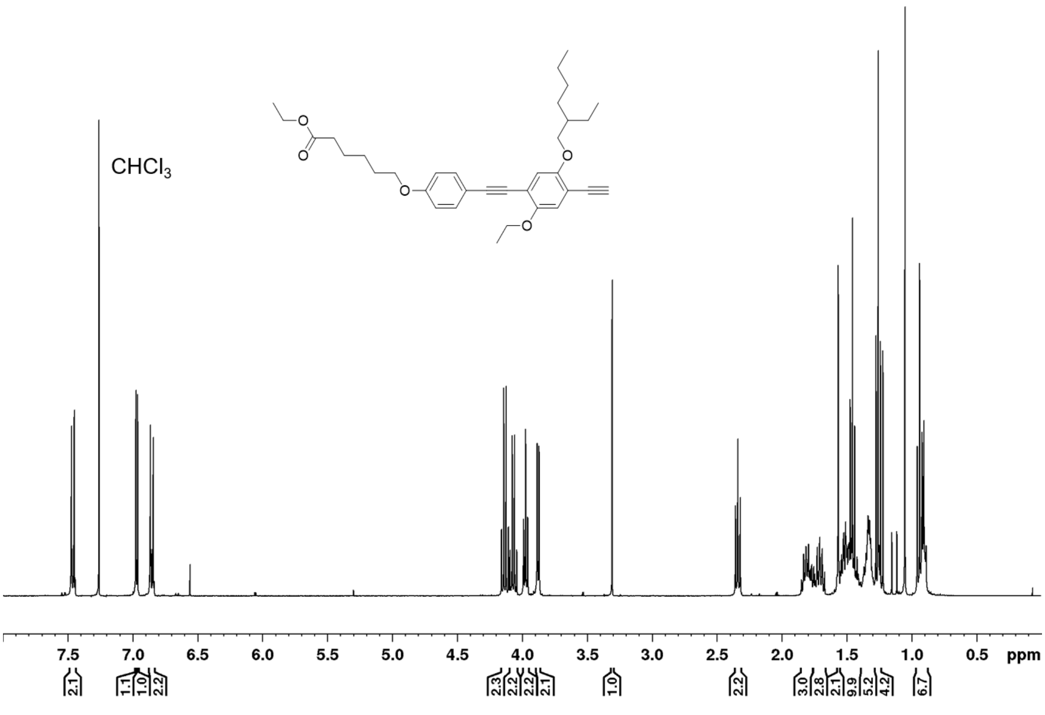

Figure S5. ^{1}H NMR spectra of **20**. Measured in $CDCl_3$, 400.1 MHz.

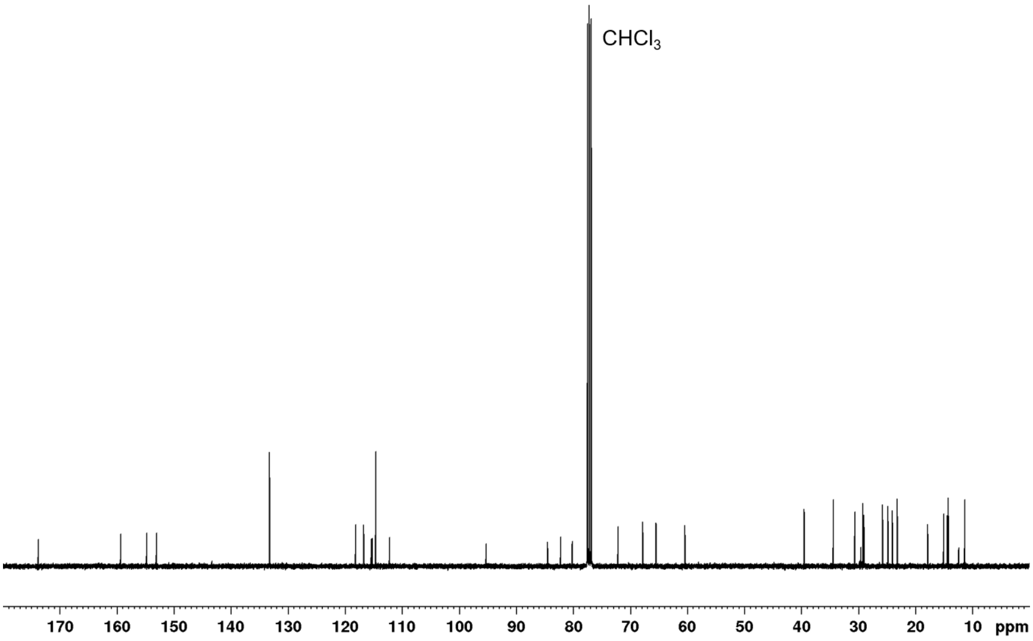

Figure S6. ^{13}C NMR spectra of **20**. Measured in $CDCl_3$, 100 MHz.

Nematogene **2**

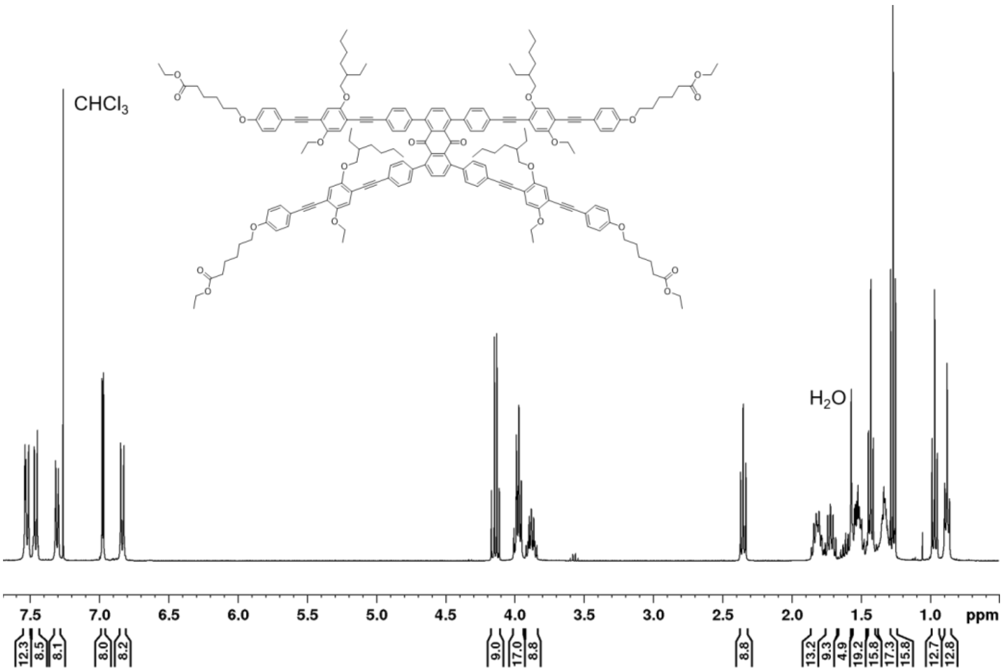

Figure S7. ^{1}H NMR spectra of **2**. Measured in $CDCl_3$, 400.1 MHz.

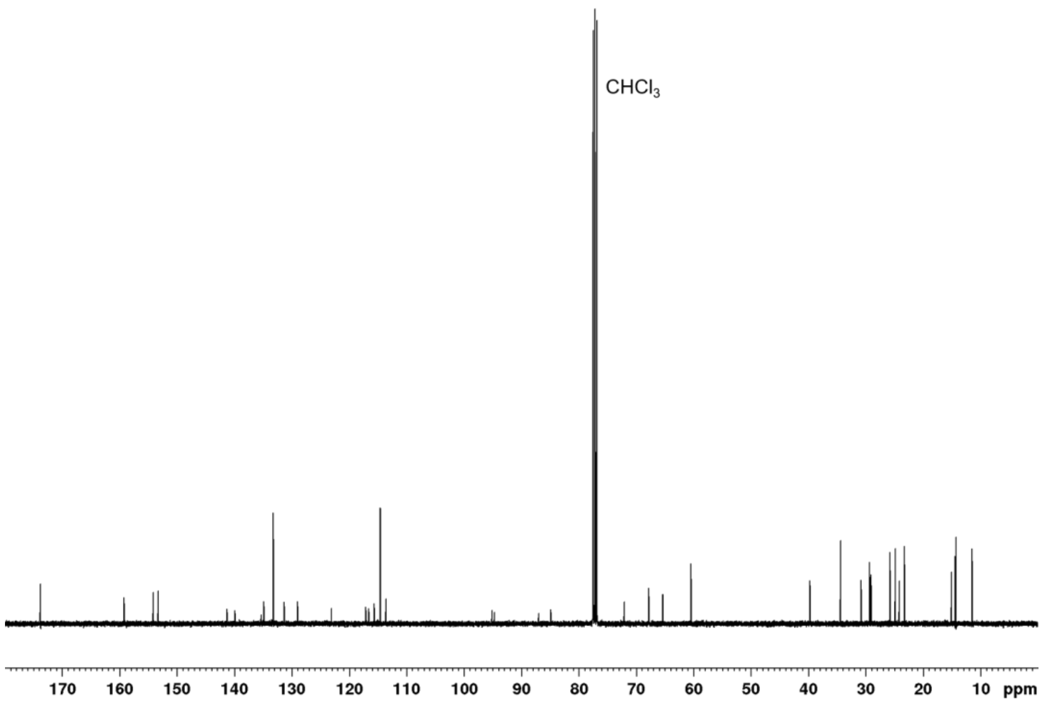

Figure S8. ^{13}C NMR spectra of **2**. Measured in $CDCl_3$, 100 MHz.

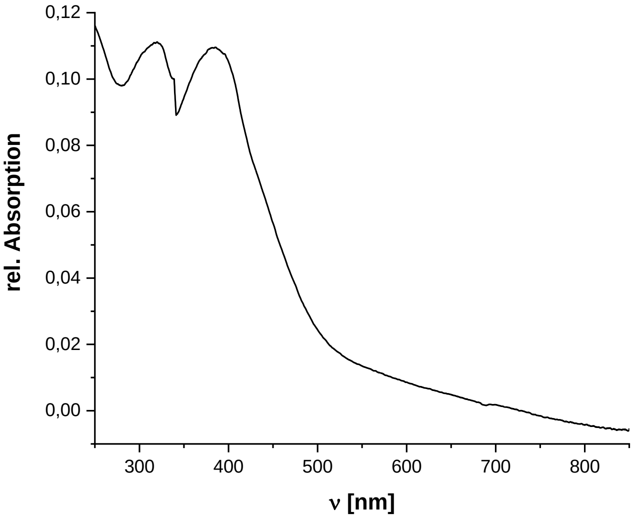

Figure S9. Solid state UV-VIS spectra of **2**. Measured as thin film on quartz glass.

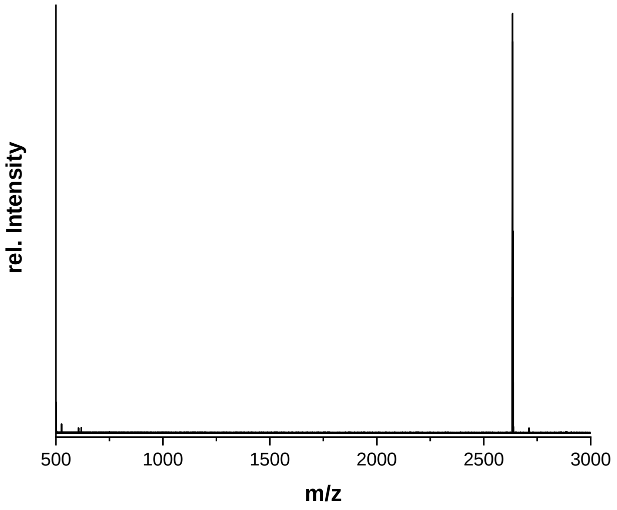

Figure S10. Mass spectra of **2**. Matrix: DCTB; Negative voltage polarity.

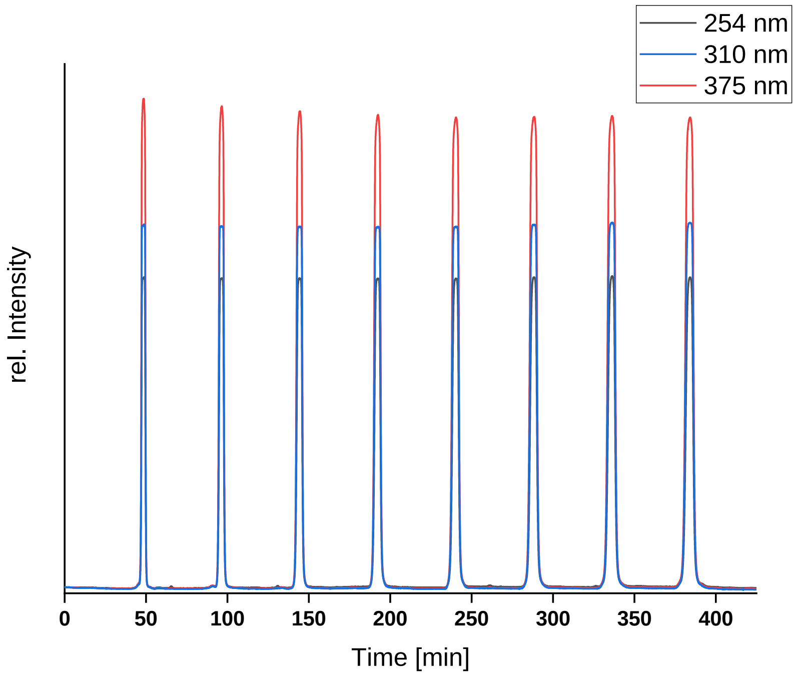

Figure S11. GPC elugram of purification process of **2** in chloroform.

5 Sample preparation

The sample were all purified by size exclusion chromatography and a subsequent freeze-drying from benzene. Since the sample slowly decomposes in the isotropic liquid at T > 250 °C, all samples were prepared at temperatures below 175 °C.

Liquid crystal cells

The LC cells for POM and TOF studies have been loaded by the capillary effect heating the sample to 170 °C.

X-ray scattering

The material have been positioned in Mark capillaries in a home-made heating block equipped with two ring magnets realizing a magnetic field of approximately 1 T. During the study the material was not heated over 180 °C. It aligned immediately when the nematic phase formed.

Cell preparation for TOF measurements

The LC sample was first filled in the homogenously aligned LC cells of thickness ≈ 8.7 µm in the isotropic phase and then cooled with a slow cooling rate of 0.5 °C/min to obtain a better molecular ordering of nematogens inside the LC cells.

6 DSC studies

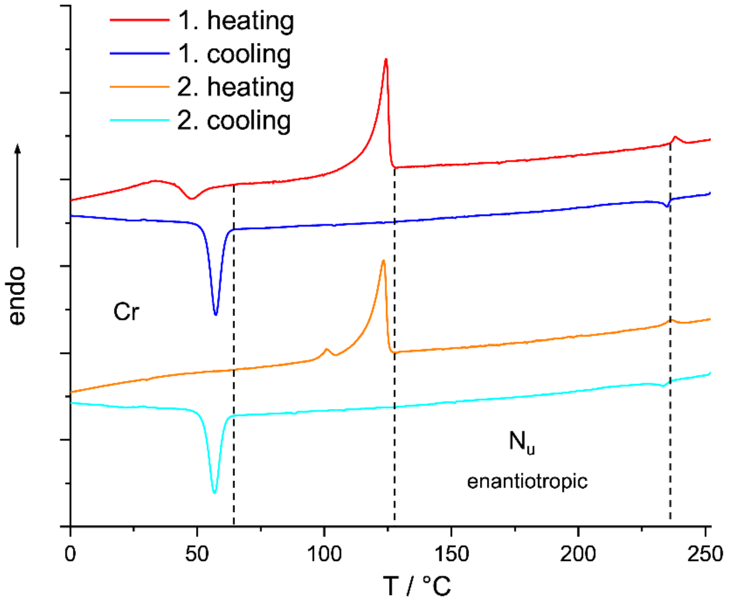

Figure S12. First and second DSC heating cycle of compound **2** at a heating rate of 10 K/min.

The second heating cycle shows less clear signals for the clearing of the substances and slightly shifted onset values for the transitions to lower temperature. This is in agreement with microscopic observations, in which the decomposition of the material has been recognized under prolonged annealing. This is the reason why all samples were prepared below 200 °C.

7 X-ray scattering

Temperature-variable X-ray scattering (XRS) studies have been performed for the sample in a 1T magnetic field. This has been achieved in a home-made sample holder between two ring magnets (*Sm2Co17 from Deutsche Techna Dr. Hahn GmbH & Co KG*). The temperature has been calibrated for this sample holder using liquid crystal calibration standards.

The studies were performed with a linear setup to cover the whole azimuth angle (sample-detector distance 13 cm, Figure 3) and for a better resolution of the wide-angle and small angle signals also with a detector tilt and a sample to detector distance of 21 cm. The latter result is highlighted in Figure S13.

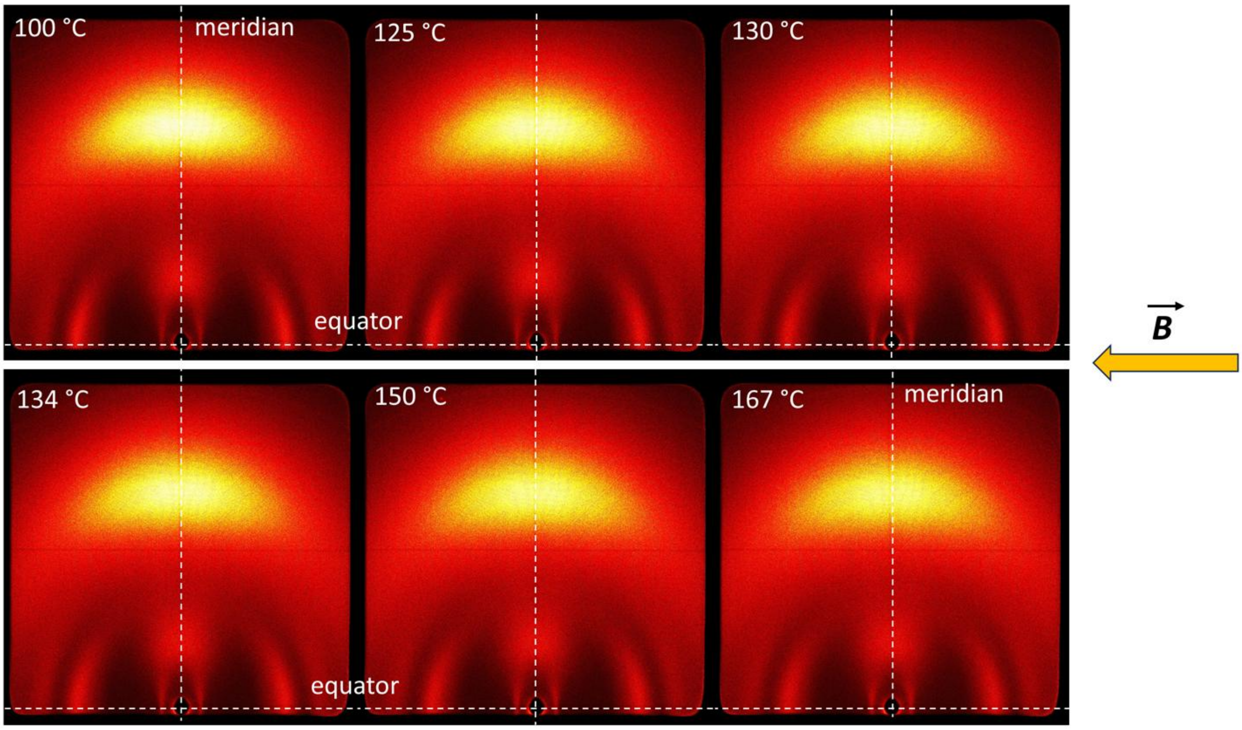

Figure S13. WAXS diffraction patterns of compound **2** recorded with a tilted detector.

The data from Figure 3F has been obtained by the evaluation of the WAXS pattern in Figure S13 with a tilted detector configuration, since the beam stop is more distant to the SAXS signal corresponding to the molecular length *L* and along the meridian the wide angle signals can be integrated completely. The patterns were calibrated with a silver behenate standard [15] using the program datasqueeze.[16] The fit of the meridional signals turned out to be most challenging, since two Lorentzians (breath *B* and the halo) were not sufficient to fit accurately the pattern. A total of four Lorentzians gave a satisfying fit consisting of a signal for the breath *B*, the mean distance between hydrocarbons d_{halo}, the mean distance along the aromatic scaffolds d_W and an additional weak signal we attribute to a periodic distance along *B* corresponding to the symmetry of the molecule analogues to the extra signal along *L* (compare the next section: simulation of the XRS pattern and Figure S16).

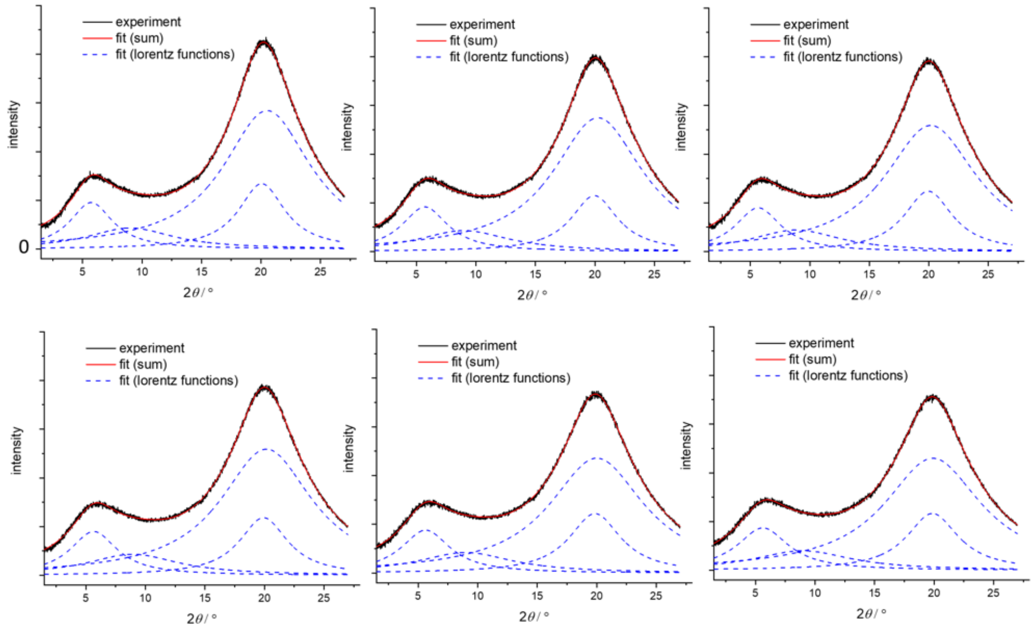

Figure S14. Integration of the WAXS patterns in Figure S13 along the meridian (experiment) and the fit functions. From Left to Right, Top: 100 °C, 125 °C, 130 °C; Bottom: 134 °C, 150 °C, 167 °C.

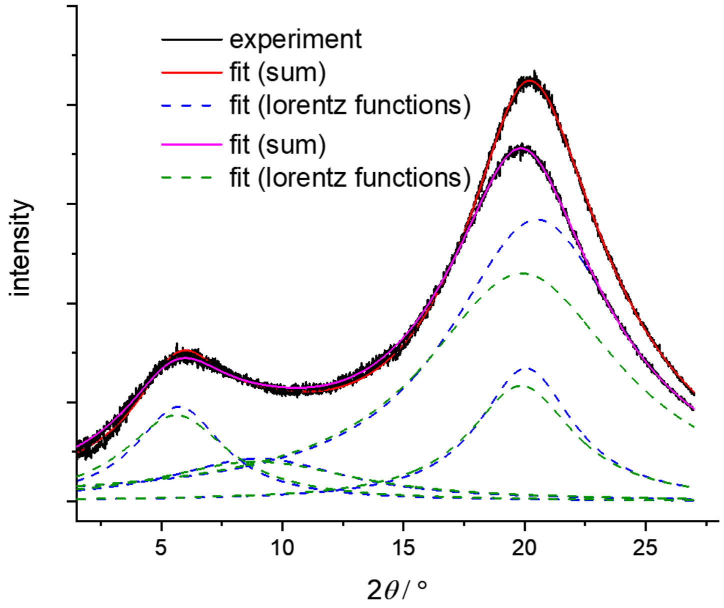

Figure S15. Comparison of the curves at 167 °C (green, magenta) and at 100 °C (blue, red).

Figure S15 shows the comparison of the WAXS intensities at 167 °C and 100 °C. With decreasing temperature the signals shift to larger angles and get more intense. This means that the packing in the nematic phase becomes more efficient. This comes along with a slight increase in the correlation length (Figure 3F) of the molecules. However, the phase remains still liquid-like, i.e. nematic.

8 Density Estimation and Calculation of the Width *W*

Density measurements by the buoyancy method

The density measurement of compound **2** were carried out by the buoyancy method adjusting a mixture of deionized water and methanol until the unknow solid sample floats. All solvents were degassed by ultrasonication.

A sample of **2** was extruded at 180 °C and cooled rapidly to ambient temperature to guaranty the formation of the glassy nematic phase. The specimens were added to a vial containing ultrasonicated methanol at 22 °C and deionized water was slowly added until the sample started to flow. The density of this liquid was subsequently determined with a pycnometer to give a density of ρ = 0.944 gcm^{-3} for the glassy nematic phase. This density value is an estimate of the minimum density because the inclusion of small gas bubbles during the extrusion process cannot completely excluded. Assuming, that the density change between 22 °C and 100 °C is based only by the temperature dependence of the aliphatic volume,[17] the minimum density at 100 °C can be estimated to be 0.917 gcm^{-3}.

Calculation of *W*

Since the molecule is roof-shaped and not flat the width *W* is not simply the distance of the molecules along the minor axis W. Knowing the base area *L* x *B* the width is calculated to be the height in which the whole molecular mass can be placed to achieve the density of 0.917 gcm^{-3}.

$\rho_{100\,°C}$ = 0.917 gcm^{-3} = 10^{-30}g Å^{-3},

L = 49.09 Å and *B* = 15.42 Å.

M =2635.43 gmol^{-1}, N_A = 6.02214*10^{23}mol^{-1}

$V_{mol} = L*B*W$

$\rho = M*N_A^{-1}*V_{mol}^{-1} = M*N_A^{-1}*L^{-1}*B^{-1}*W^{-1}$

$W = \rho^{-1}*M*N_A^{-1}*L^{-1}*B^{-1}$ = (6.31 ± 0.47) Å

The error ΔW was calculated assuming $\Delta\rho$ = ± 0.05 gcm^{-3}, ΔL = ± 0.2 Å, ΔB = ± 0.2 Å.

9 DFT calculation, MD Simulation and XRS simulation

Electronic Structure Calculations

Electronic structure calculations were performed at the B3LYP/6-31G(d) level of DFT[18] using the Gaussian G09 revision d01 software package.[19] Following geometry optimisation we performed a frequency calculation to assess the validity of the resulting geometry.

Molecular Dynamics Simulations

Fully atomistic molecular dynamics (MD) simulations were performed in Gromacs 2019.2 with GPU acceleration.[20] Materials were modelled using the General Amber Force Field (GAFF).[21] Atomic charges were determined using the RESP method[22] for geometries optimised at the B3LYP/6-31G(d) level of DFT[18] using the Gaussian G09 revision d01 software package.[19] Topologies were generated using AmberTools 16[23] and converted into Gromacs readable format with Acpype.[24]

We initially constructed a low density lattice of 400 molecules of with random positional and orientational order. Following energy minimization by the steepest decent method we performed short (25 ns) equilibration simulations in the NVE and NVT (T = 700 K) ensembles. We then performed a short 'compression' simulation (25 ns) at 600K with an isotropic barostat (P = 100 Bar) to compress the simulation to a liquid like density (~ 1.05 g cm^3). The resulting isotropic configurations were then used as a starting configuration for further simulations. For production MD simulations we used an anisotropic barostat with a pressure of 1 Bar and at the indicated temperatures. Simulations were performed for in excess of 2 µs, unless otherwise noted.

Simulations employed periodic boundary conditions in xyz. Simulations employed a time step of 2 fs, with trajectory data recorded every 5000 steps. Bonds lengths were constrained to their equilibrium values with the LINCS algorithm.[25] During production MD simulations the system pressure was maintained using an anisotropic Parrinello-Rahamn barostat.[26] Simulation temperature was controlled with a Nosé–Hoover thermostat.[27] Compressabilities in xyz dimensions were set to 4.5e-5, with the off-diagonal compressibilities were set to zero to ensure the simulation box remained rectangular. Long-range electrostatic interactions were calculated using the Particle Mesh Ewald method with a cut-off value of 1.2 nm. A van der Waals cut-off of 1.2 nm was used. MD trajectories were visualised using PyMOL 4.5. Q-tensor analysis was performed using MDTraj 1.9.8.[28]

We judged a simulation to be 'nematic' when the second-rank orientational order parameter (<*P*2>) takes a value of > 0.3, and conversely we judge it isotropic if <*P*2> is below this value. We calculate <P_2> *via* the Q-tensor according to equation (1);

$$Q_{\alpha\beta} = \frac{1}{N}\sum_{m=1}^{N}\frac{3a_{m\alpha}a_{m\beta}-\delta_{\alpha\beta}}{2} \qquad (1)$$

where N is the number of monomers, m is the monomer number within a given simulation, α and β represent the Cartesian x, y and z axes, delta is the Kronecker delta, *a* is a vector that describes the molecular long axis, which is computed for each monomer as the eigenvector associated with the smallest eigenvalue of the inertia tensor. The director at each frame was defined as the eigenvector associated with the largest eigenvalue of the ordering tensor. The order parameter <*P2*> corresponds to the largest eigenvalue of $Q_{\alpha\beta}$. The biaxial order parameter <*B*> is defined here as the difference between the two smallest eigenvalues of $Q_{\alpha\beta}$.

Fully atomistic molecular dynamics (MD) simulations of **1** were performed with a view to reconstructing the experimental wide-angle X-ray diffraction pattern shown in Figure 3a-d. The first step in this process is to obtain a suitable nematic-like phase; fortuitously, compound **1** readily forms a stable nematic phase with an orientational order parameter of 0.66+/-0.07 and a biaxiality parameter B = 0.092+/-0.008 at 373 K (100 °C). A representative instantaneous configuration is shown in Figure S16.

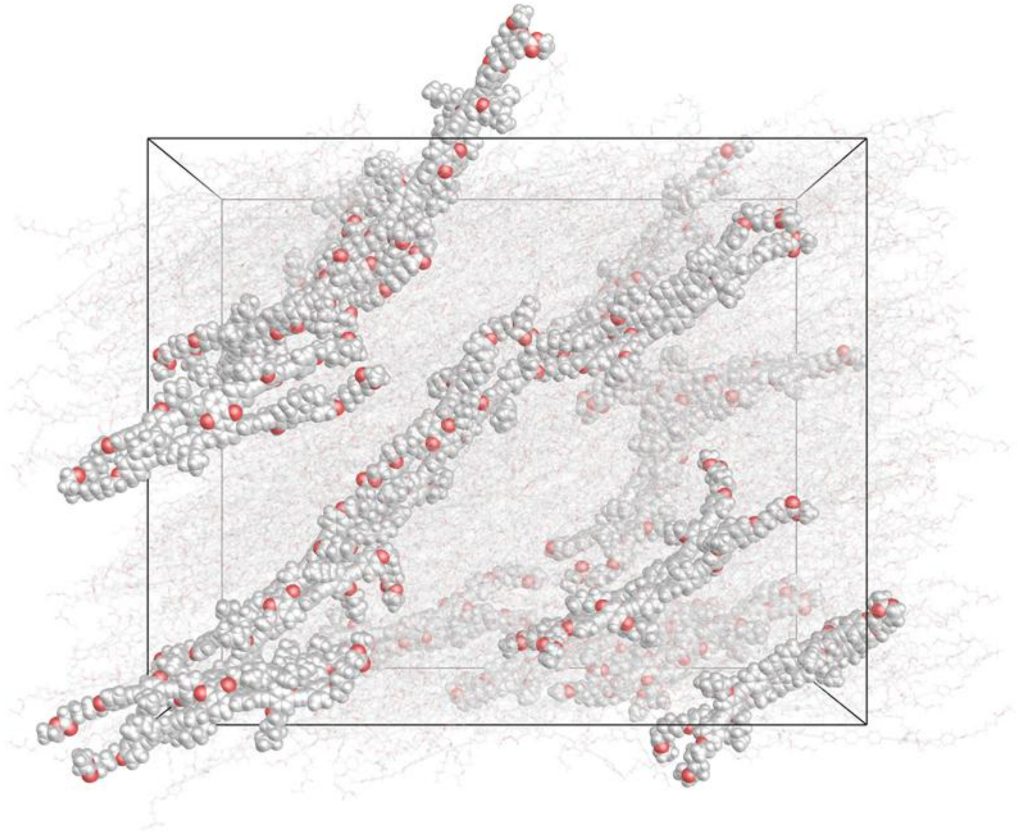

Figure S16. Instantaneous configuration of the molecular dynamics simulation of **1** at a temperature of 373 K at 500 ns. Molecules are shown as wireframe, with 18 randomly selected molecules shown as space filling models. The periodic boundaries of the simulation are shown with solid black lines.

Simulation of Small Angle X-ray Scattering Patterns

We simulated X-ray scattering from MD trajectory from the complex 3D structure factor using in house developed Python tools which exploit Numba JIT compilation; code is available at https://github.com/RichardMandle/simusaxs

We use MDTraj 1.9.8.[27] to handle trajectory I/O operations, and to orient the trajectory such that the nematic director in a given frame was aligned with the x-axis of the simulation; this mimics real world experiments in which an external magnetic field aligns the nematic director is a vector approximately perpendicular to the incident radiation.

For each frame in the trajectory we map the electron density onto a real-space grid whose extent ultimately defines the resolution of the resultant 2D SAXS pattern. A hamming window function is applied to the electron density grid, which is subsequently zero-padded and finally transformed to the complex 3D structure factor *via* a 3D fast Fourier transform and shifting the zero-frequency component to the centre. The 3D structure factor is interpolated using the RegularGridInterpolator function of scipy, and this is also used to clip data to the desired Q-range. Finally, summation of the real part of the interpolated 3D structure factor along a given axes yields the simulated SAXS pattern in the corresponding plane; e.g. summation about x- gives the yz plane.

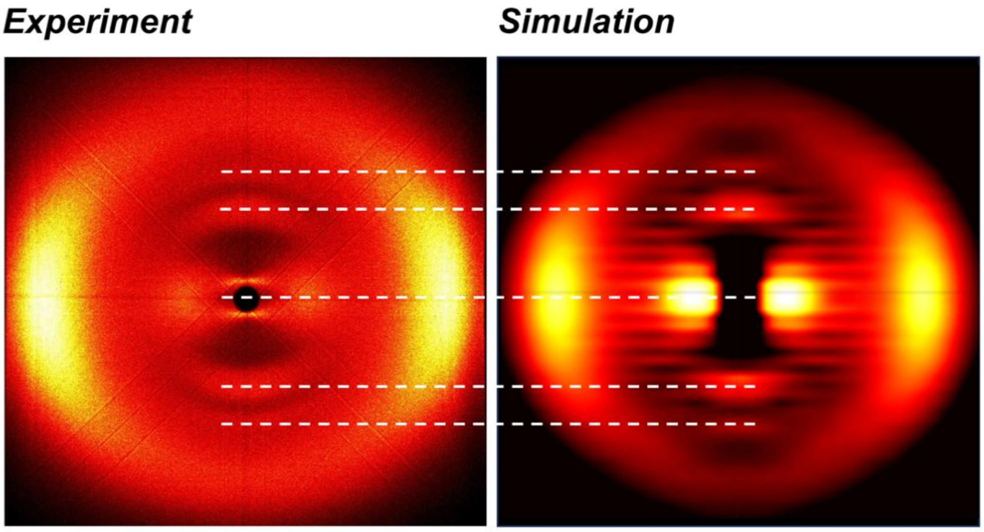

Figure S17. Experimental and simulated wide-angle X-ray diffraction pattern of lead compound **1**.

The simulated XRS pattern obtained from an MD clearly shows that the diffuse signals generated by the molecular long axis *L* can be reproduced. Owing to the way the FFT data is windowed and shifted we lose information about scattering at the smallest angles. This has been earlier interpreted as a consequence of next neighbour positions in the nematic phase and the symmetry of the nematogen with a intramolecular periodic electron density distribution.

10 Time-of-flight (TOF) charge carrier mobility

Cell preparation for TOF measurements: The LC sample was first filled in the homogenously aligned LC cells of thickness ≈ 8.7 µm in the vacuum oven in the isotropic phase and then cooled with a slow cooling rate of 0.5 °C/min to obtain a better molecular ordering of nematogens inside the LC cells. Subsequently, the cell was sealed with the UV sealant NOA68 (Norland) to avoid the leakage of LC material at higher temperatures.

Time-of-Flight measurement: In the time-of-flight (ToF) technique, N_2 pulsed laser equipped with a 337 nm excitation wavelength was used. The LC compound was irradiated by laser pulse and the displacement of electrons was recorded using a digital oscilloscope (Keysight, DSOX3022T) under the application of an external negative voltage between -20 and -30 V. For the temperature-dependent TOF measurements, the temperature was controlled by the Linkam LTS 350 hot plate connected to a temperature controller Linkam TMS 94 with an accuracy of ± 1 °C. The output transient photocurrent curves were used to calculate the electron mobility (μ_e) by using the formula $\mu_e = \frac{d^2}{V\tau}$, where τ is the transit time obtained by photocurrent curves, V is the applied voltage and d is the thickness of LC cells.

Transient times: For each voltage the results of the current-time transients were transformed in a normalized double logarithmic representation (see for example Figure S18).[29,30] The subsequent tangents could be positioned with the intersection marking the transient time.

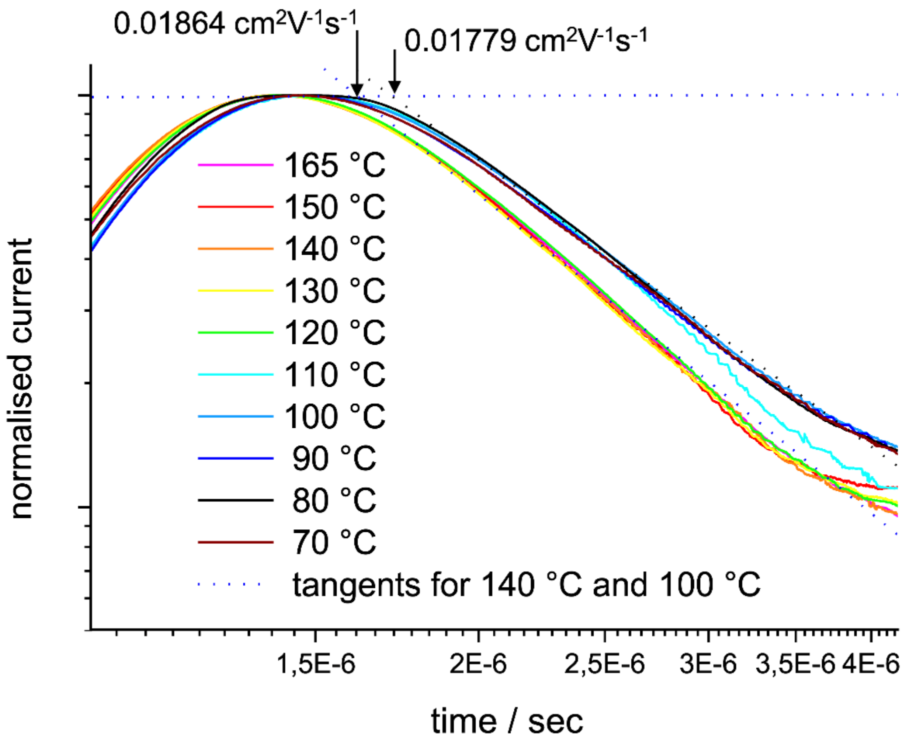

Figure S18. TOF normalized double logarithmic plots at -25 V.

11 Second Harmonic Generation (SHG)

The TOF LC sample was used for the SHG study at tilted incident. Figure S19 highlights the results. A weak signal increases with the increasing electric field. The weak signal points to the presence of small polar aggregates.

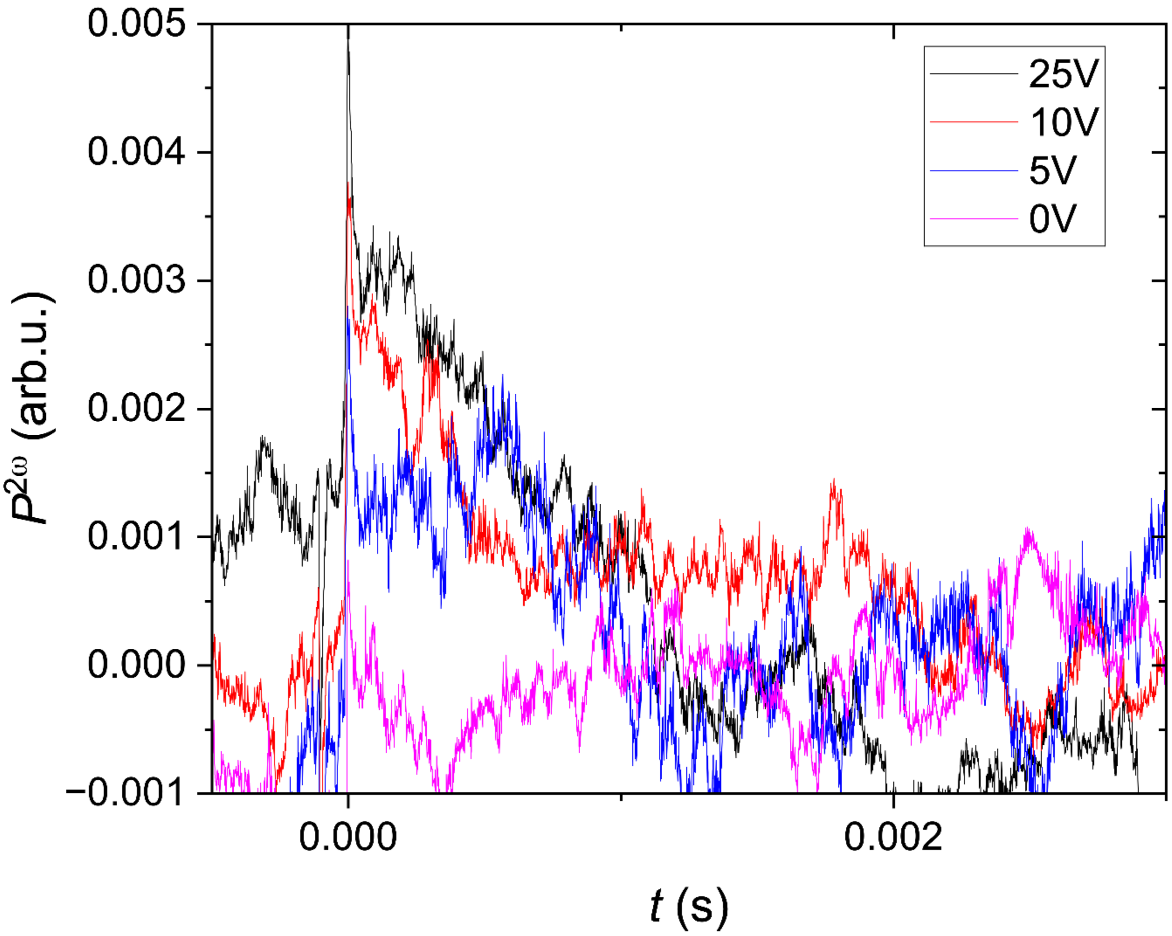

Figure S19. E-field dependent SHG signal of compound **1** at 150 °C.

12 References

[1] E. R. Barth, D. Längle, F. Wesseler, C. Golz, A. Krupp, D. Schade, C. Strohmann, *Eur. J. Inorg. Chem.* **2019**, *2020*, 176-181.

[2] K. Fuchibe, T. Akiyama, *J. Am. Chem. Soc.* **2006**, *128*, 1434-1435.

[3] T. Thiemann, Y. Tanaka, J. Iniesta, *Molecules* **2009**, *14*, 1013-1031.

[4] X. Feng, J. Wu, V. Enkelmann, K. Müllen, *Org Lett* **2006**, *8*, 1145-1148.

[5] R. Edwards, W. de Vries, A. D. Westwell, S. Daniels, T. Wirth, *Eur. J. Org. Chem.* **2015**, 6909-6916.

[6] T. Kusumi, T. Ooi, M. R. Walchli, H. Kakisawa, *J. Am. Chem. Soc.* **2002**, *110*, 2954-2958.

[7] P. M. Paduraru, R. T. Popoff, R. Nair, R. Gries, G. Gries, E. Plettner, *J. Comb. Chem.* **2008**, *10*, 123-134.

[8] J. I. Andrés-Gil, J. M. Bartolomé-Nebreda, R. M. Alvarez-Escobar, M. H. M. Bakker, A. A. H. P. Megens, World Intellectual Property Organization, WO 2004/016621 A1, **2004**.

[9] S. H. Boyer, H. Jiang, J. D. Jacintho, M. V. Reddy, H. Li, W. Li, J. L. Godwin, W. G. Schulz, E. E. Cable, J. Hou, R. Wu, J. M. Fujitaki, S. J. Hecker, M. D. Erion, *J. Med. Chem.* **2008**, *51*, 7075-7093.
[10] X. Zhu, K. N. Plunkett, *J. Org. Chem.* **2014**, *79*, 7093-7102.
[11] T. A. Cross, M. C. Davis, *Synth. Commun.* **2008**, *38*, 499-516.
[12] S. Hoger, K. Bonrad, A. Mourran, U. Beginn, M. Moller, *J Am Chem Soc* **2001**, *123*, 5651-5659.
[13] M. Desroches, J.-F. Morin, *RSC Advances* **2017**, *7*, 17117-17121.
[14] M. Lehmann, J. Seltmann, *Beilstein J. Org. Chem.* **2009**, *5*, 73.
[15] T. C. Huang, H. Toraya, T. N. Blanton, Y. Wu, *J. Appl. Cryst.* **1993**, *26*, 180-184.
[16] P. Heiney, data squeeze 3.0.16, https://www.physics.upenn.edu/~heiney/datasqueeze
[17] B. Donnio, B. Heinrich, H. Allouchi, J. Kain, S. Diele, D. Guillon, D. W. Bruce, *J. Am. Chem. Soc.* **2004**, *126*, 15258.
[18] a) C. Lee, W. Yang, R. G. Parr, *Phys. Rev. B* **1988**, *37*, 785-789; b) A. D. Becke, *J. Chem. Phys.* **1993**, *98*, 5648-5652.
[19] M. J. Frisch, G. W. Trucks, H. B. Schlegel, G. E. Scuseria, M. A. Robb, J. R. Cheeseman, G. Scalmani, V. Barone, B. Mennucci, G. A. Petersson, H. Nakatsuji, M. Caricato, X. Li, H. P. Hratchian, A. F. Izmaylov, J. Bloino, G. Zheng, J. L. Sonnenberg, M. Hada, M. Ehara, K. Toyota, R. Fukuda, J. Hasegawa, M. Ishida, T. Nakajima, Y. Honda, O. Kitao, H. Nakai, T. Vreven, J. A. Montgomery Jr., J. E. Peralta, F. Ogliaro, M. J. Bearpark, J. Heyd, E. N. Brothers, K. N. Kudin, V. N. Staroverov, R. Kobayashi, J. Normand, K. Raghavachari, A. P. Rendell, J. C. Burant, S. S. Iyengar, J. Tomasi, M. Cossi, N. Rega, N. J. Millam, M. Klene, J. E. Knox, J. B. Cross, V. Bakken, C. Adamo, J. Jaramillo, R. Gomperts, R. E. Stratmann, O. Yazyev, A. J. Austin, R. Cammi, C. Pomelli, J. W. Ochterski, R. L. Martin, K. Morokuma, V. G. Zakrzewski, G. A. Voth, P. Salvador, J. J. Dannenberg, S. Dapprich, A. D. Daniels, Ö. Farkas, J. B. Foresman, J. V. Ortiz, J. Cioslowski, D. J. Fox, *Gaussian 09* **2009**.
[20] a) H. J. C. Berendsen, D. van der Spoel, R. van Drunen, *Computer Physics Communications* **1995**, *91*, 43-56; b) E. Lindahl, B. Hess, D. van der Spoel, *Molecular modeling annual* **2001**, *7*, 306-317; c) D. Van Der Spoel, E. Lindahl, B. Hess, G. Groenhof, A. E. Mark, H. J. Berendsen, *Journal of computational chemistry* **2005**, *26*, 1701-1718; d) B. Hess, C. Kutzner, D. van der Spoel, E. Lindahl, *Journal of Chemical Theory and Computation* **2008**, *4*, 435-447; e) S. Pronk, S. Páll, R. Schulz, P. Larsson, P. Bjelkmar, R. Apostolov, M. R. Shirts, J. C. Smith, P. M. Kasson, D. van der Spoel, B. Hess, E. Lindahl, *Bioinformatics (Oxford, England)* **2013**, *29*, 845-854; f) M. J. Abraham, T. Murtola, R. Schulz, S. Páll, J. C. Smith, B. Hess, E. Lindahl, *SoftwareX* **2015**, *1-2*, 19-25; g) S. Páll, M. J. Abraham, C. Kutzner, B. Hess, E. Lindahl, in *Solving Software Challenges for Exascale* (Eds.: S. Markidis, E. Laure), Springer International Publishing, Cham, **2015**, pp. 3-27.
[21] J. Wang, R. M. Wolf, J. W. Caldwell, P. A. Kollman, D. A. Case, **2004**, *25*, 1157-1174.
[22] C. I. Bayly, P. Cieplak, W. Cornell, P. A. Kollman, *J. Phys. Chem.* **1993**, *97*, 10269-10280.
[23] a) D. A. Case, T. E. Cheatham Iii, T. Darden, H. Gohlke, R. Luo, K. M. Merz Jr, A. Onufriev, C. Simmerling, B. Wang, R. J. Woods, *J. Comp. Chem.* **2005**, *26*, 1668-1688; b) J. Wang, W. Wang, P. A. Kollman, D. A. Case, *J. Mol. Graph. Model.* **2006**, *25*, 247-260.
[24] A. W. Sousa da Silva, W. F. Vranken, *BMC Research Notes* **2012**, *5*, 367.
[25] B. Hess, H. Bekker, H. J. C. Berendsen, J. G. E. M. Fraaije, *J. Comp. Chem.* **1997**, *18*, 1463-1472.
[26] a) M. Parrinello, A. Rahman, *J. Appl. Phys.* **1981**, *52*, 7182-7190; b) S. Nosé, M. L. Klein, *Mol. Phys.* **1983**, *50*, 1055-1076.
[27] a) S. Nosé, *Mol. Phys.* **1984**, *52*, 255-268; b) W. G. Hoover, *Phys. Rev. A* **1985**, *31*, 1695-1697.
[28] R. T. McGibbon, K. A. Beauchamp, M. P. Harrigan, C. Klein, J. M. Swails, C. X. Hernández, Christian R. Schwantes, L.-P. Wang, T. J. Lane, V. S. Pande, *Biophys. J.* **2015**, *109*, 1528-1532.

[29] R. L. Lidberg, Time-of-Flight Investigation of Charge Carrier Mobilities in Oligoacene Single Crystals, University of Minnesota **2017**.
[30] T. J. Pundsack, N. O. Haugen, L. R. Johnstone, C. D. Frisbie, R. L. Lidberg, *Appl. Phys. Lett.* **2015**, *106*, 113301
